# Evidence of selection for an accessible nucleosomal array in human

**DOI:** 10.1186/s12864-016-2880-2

**Published:** 2016-07-29

**Authors:** Guénola Drillon, Benjamin Audit, Françoise Argoul, Alain Arneodo

**Affiliations:** 1 Univ Lyon, Ens de Lyon, Univ Claude Bernard Lyon 1, CNRS, Laboratoire de Physique, Lyon, F-69342 France; 2 LOMA, Université de Bordeaux, CNRS, UMR 5798, 51 Cours de le Libération, Talence, F-33405 France

**Keywords:** Nucleosome depleted regions, Sequence evolution, Nucleosome ordering, Mutation rates, GC content, Human genome

## Abstract

**Background:**

Recently, a physical model of nucleosome formation based on sequence-dependent bending properties of the DNA double-helix has been used to reveal some enrichment of nucleosome-inhibiting energy barriers (NIEBs) nearby ubiquitous human “master” replication origins. Here we use this model to predict the existence of about 1.6 millions NIEBs over the 22 human autosomes.

**Results:**

We show that these high energy barriers of mean size 153 bp correspond to nucleosome-depleted regions (NDRs) in vitro, as expected, but also in vivo. On either side of these NIEBs, we observe, in vivo and in vitro, a similar compacted nucleosome ordering, suggesting an absence of chromatin remodeling. This nucleosomal ordering strongly correlates with oscillations of the GC content as well as with the interspecies and intraspecies mutation profiles along these regions. Comparison of these divergence rates reveals the existence of both positive and negative selections linked to nucleosome positioning around these intrinsic NDRs. Overall, these NIEBs and neighboring nucleosomes cover 37.5 % of the human genome where nucleosome occupancy is stably encoded in the DNA sequence. These 1 kb-sized regions of intrinsic nucleosome positioning are equally found in GC-rich and GC-poor isochores, in early and late replicating regions, in intergenic and genic regions but not at gene promoters.

**Conclusion:**

The source of selection pressure on the NIEBs has yet to be resolved in future work. One possible scenario is that these widely distributed chromatin patterns have been selected in human to impair the condensation of the nucleosomal array into the 30 nm chromatin fiber, so as to facilitate the epigenetic regulation of nuclear functions in a cell-type-specific manner.

**Electronic supplementary material:**

The online version of this article (doi:10.1186/s12864-016-2880-2) contains supplementary material, which is available to authorized users.

## Background

To better understand the function and evolution of eukaryotic genomes, it is of prime importance to decipher to which extent the different steps of DNA compaction, from the nucleosomal primary DNA structure to the chromatin loop tertiary DNA structure, have been encoded in the DNA sequence during evolution [1, 2]. Nowadays, it is well recognized that the dynamics of DNA folding and unfolding within living cells plays an important role in the regulation of nuclear functions [3]. Transcription, replication and the associated repair mechanisms are known to underlie the increasing complexity of neutral mutation patterns in mammalian genomes [4]. In human, all substitution rates increase monotonously with replication timing, as an indication of some weakening in the fidelity of the replication machinery during the S phase [5, 6]. Over evolutionary time-scales, transcription and replication have been shown to induce strand-specific asymmetry in mutations [1, 7]. Because chromatin structure and dynamics vary across the genome, one can expect that chromatin influences the course of sequence evolution over evolutionary time and contributes to the observed regional variations in the mutational landscape. Recent studies have revealed rather contrasting situations depending on whether DNA accessibility has a greater impact on mutagenic or repair processes. Lower (resp. higher) substitution frequency is generally observed in open (resp. close) chromatin environment [8]. At a fine scale, the nucleosome was early recognized as being at the heart of the trade-off between the necessity of compacting DNA in the cell nucleus and the required accessibility to regulatory proteins [1, 3]. Recent studies of sequence evolution in various eukaryotic organisms have confirmed the existence of some correlation between sequence divergence and nucleosome positioning. In yeast, the overall levels of divergence were observed to be higher near the nucleosome cores than in the linker regions [9, 10] and interpreted as the consequence of the limited access of nucleosomal DNA to DNA repair proteins [11]. Alternative explanations have been proposed, invoking purifying selection in linker regions in particular to maintain nucleosome-disfavoring codons in genic regions and nucleosome-antipositioning sequences at regulatory regions like gene promoters and enhancers [9, 12–14]. A similar observation was reported in medaka fish [15]. Independant analysis of the first available genome-wide nucleosome maps in human [16–18] have confirmed that, in mammals like in yeast, single nucleotide polymorphisms (SNPs) are enriched around the position of the nucleosome cores and depleted in the linker regions. By combining the analysis of interspecies and intraspecies divergence rates, Prendergast and Semple [18] have further provided evidence for selection likely acting on particular base substitutions to maintain some optimum GC composition in both core (high GC content) and linker (low GC content) DNA. By showing that these signatures of selection concern non-coding as well as coding sequences, in bulk as well as in epigenetically modified nucleosomes, these authors have provided some very promising understanding of the important role played by the DNA sequence in nucleosome positioning. These signatures of selection were derived from a relatively small subset of well-positioned nucleosomes that cover about 5 % of the human genome. If this coverage is comparable to protein coding genes coverage, one may wonder whether the nucleosomal 1D organization of the chromatin fiber has a more widespread impact on the divergence of regions traditionally considered as evolving neutrally, including intronic and intergenic sequences.

The role of the genomic sequence on nucleosome positioning in vivo has become an important issue [19–22]. It was early recognized that the periodic occurence of dinucleotides with a period of 10−11 bp favors the formation of nucleosomes [23–26]. However, in *S. cerevisiae*, Drosophila and mammals, this periodicity was shown to account for no more than 20 % of in vivo nucleosome positioning above what is expected by chance [19, 27, 28] and it is almost invisible in the human genome after masking the recently integrated Alu-sequences [29]. An alternative to the tight histone binding obtained with favorable positioning sequences is the statistical positioning of nucleosomes nearby nucleosome inhibitory energy barriers (NIEBs), that can be encoded either via unfavorable sequences that potentially resist to the structural distortions required by nucleosome formation or particular sequences that may recruit transcription factors, or/and other protein complexes such as chromatin regulators that may compete with the nucleosomes [19, 21, 22]. Due to the interplay between boundary confinement and nucleosome-nucleosome excluded volume interactions, some statistical short-range ordering can establish near a NIEB, and gradually disappear away from the barrier [19, 30–33]. In that context, a simple physical model of nucleosome assembly based on the computation of the free energy cost of bending a DNA fragment of a given sequence from its natural curvature to the final superhelical structure around the histone core, was recently shown to mimic in vitro nucleosome occupancy data remarkably well [19, 34, 35]. When compared to in vivo data in *S. cerevisiae* and *C. elegans*, this sequence-dependent thermodynamical model performs as well as models based on statistical learning, suggesting that in these organisms, the in vivo nucleosome array organization is, to a large extent, controlled by the underlying genomic sequence, although it is also subject to the finite-range remodeling action of external factors.

A key issue to the understanding of chromatin-mediated regulation of nuclear functions is the relative positioning of regulatory sites with respect to NIEBs encoded in the DNA sequence. Active regulatory sequences are commonly thought to lie in accessible chromatin regions. This was verified in vivo in eukaryotic genomes but not necessarily in vitro, as recently noticed in *Arabidopsis* [36], Drosophila [37], mouse [37–40] and human [41, 42]. In *S. cerevisiae*, nucleosome-depleted regions (NDRs) were mainly observed in vivo at transcription start sites (TSS), transcription termination sites (TTS) [31, 41, 43–45] and at active DNA replication origins [46, 47]. Interestingly, most of these NDRs correspond to intrinsic NDRs encoded in the DNA sequence, up to some shape remodeling and phasing. If similar sequence-driven NDR regulation of transcription and replication initiation is likely to operate in different yeast species [48–50] and possibly in *C. elegans* [51], the situation appears to be different in mammals, where regulatory sequences were shown to have higher-than-average intrinsic nucleosome occupancy. Unlike in yeast where genes are mostly constitutively expressed and thus would be expected to maintain low GC promoters, most human promoters have a high GC content, that indeed corresponds to nucleosome preference in vitro [41, 42] and reduced frequency of rigid poly(dA:dT) sequences that impair nucleosome formation and favor nucleosome disassembly [23, 31, 52–55]. Recent experimental and bioinformatics studies in the mouse genome [37, 40] have confirmed that nucleosomes are intrinsically preferentially positioned at replication origins and removed when actived, as previously observed in *Arabidopsis* [36]. Altogether, these recent results suggest that, in higher eukaryotes, a high nucleosome affinity is directly programmed at regulatory sequences to intrinsically restrict access to regulatory information that will mostly be used in vivo in an epigenetically-controlled cell-type-dependent manner [41]. This probably explains that so far, only little attention has been paid to intrinsic NDRs in mammal chromatin.

Here we analyze in vitro and in vivo nucleosome occupancy data in human, using as a reference the set of NIEBs predicted by the physical model based on sequence-dependent DNA bending properties [19, 34, 35]. We show that these intrinsic NDRs, together with the flanking nucleosomes, are actually encoded in the GC content and are widely spread over the 22 human autosomes, covering about 37.5 % of the genome. When further investigating interspecies and intraspecies rates of divergence, we bring significant evidence of selection pressure to maintain both an optimal GC content at flanking nucleosomes and a robust GC content depletion in NDRs relative to the local bulk GC content. We comment on the observed deviations from neutral evolution as a possible indication of the selection of an open, accessible and dynamic 10 nm chromatin fiber to constitutively facilitate in each cell-type the epigenetic regulation of nuclear functions.

## Results

### Intrinsic NIEBs correspond to in vitro and in vivo NDRs

When running the physical model based on sequence-dependent DNA bending properties (Methods) over the 22 human autosomes, we predicted a total of 1,581,256 NIEBs of width ranging from 36 to 450 bp with a mean size of 153 bp (Additional file 1: Figure S1) comparable to the mean size of the NDRs observed in the nucleosome occupancy experimental data [56, 57]. As expected, the histogram of GC content computed in the NIEBs is significantly (*P*≪10^−3^, see Methods) shifted towards lower values ($\overline {\text {GC}}=26.5$ %, Additional file 1: Figure S2B) as compared to the histogram obtained genome-wide ($\overline {\text {GC}}=37.9$ %, Additional file 1: Figure S2A) and to the histogram computed over the 300 bp flanking regions on the left and the right of these NIEBs ($\overline {\text {GC}}=42.5$ %, Additional file 1: Figure S2C). Hence NIEBs correspond to AT-rich regions likely containing rigid poly(dA:dT) tracks known to be important determinants of nucleosome depletion in vitro as well as in vivo. This is consistent with the observation that these regions also resist to DNase I cleavage and on average are less sensitive to DNase I than the genome average (normalized DNase I sensitivity raw tag density =0.82, *P*≪10^−3^).

**Fig. 1:**
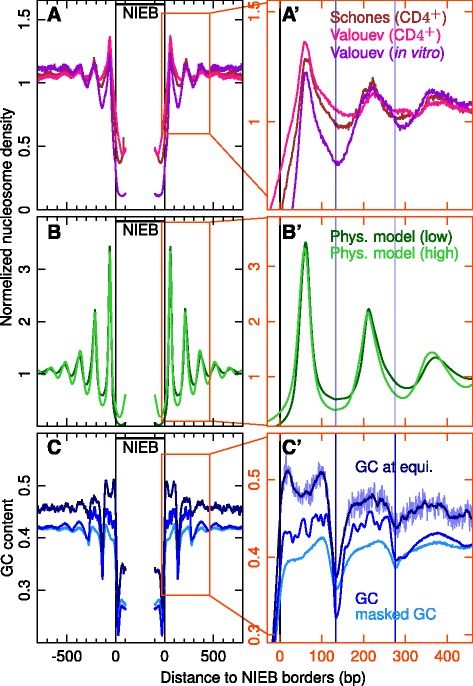
Normalized (with respect to genome average) mean nucleosome density on both sides of the 1,581,256 NIEBs predicted by the sequence-dependent physical model. **a** “Schones” in vivo (*brown*), “Valouev” in vivo (*pink*) and “Valouev” in vitro (*purple*) data (Methods). **b** Numerical mean profiles predicted by the physical model at low (*dark green*) and high (*light green*) genomic nucleosome coverages (Methods). **c** Mean GC content (*blue*), repeat-masked GC content (*sky blue*) and GC content at equilibrium (*navy blue*). **a**’,**b**’,**c**’ are zooms on the profiles in (**a**,**b**,**c**) on the right-hand side of the in silico NIEBs ; *vertical blue lines* correspond to local minima of the GC content (they will be reported in Figs. 4, 5, 6 and 7 for comparison). All profiles were computed at 1 bp resolution, except the ‘GC at equilibrium’ which was smoothed over 10 bp windows (its non-smoothed profile is shown as a background in (**c**’))

When investigating nucleosome density inside these energetically unfavourable regions, we confirmed that they correspond to NDRs as compared to genome average (Fig. 1a) and this not only for “Valouev” in vitro data (normalized nucleosome tag density = 0.15) but also for “Valouev” in vivo data (0.60) and “Schones” in vivo data (0.44) (Methods). Given the variability in sequencing depth in nucleosome MNase-seq data [42, 57], we tested whether the observed “mean” depletion reflects MNase-seq profile across individual NIEBs by comparing nucleosome tag densities within the barriers and in the two 300 bp flanking windows (Methods). We first excluded from our analysis the 43,364 NIEBs and flanking windows (2.7 % of our sample) lacking sufficient mappability to perform the test (Methods). Then, from this comparison, for each MNase-seq data set, we classified the 1,537,892 mappable NIEBs into 3 groups (Methods): (G1) NIEBs significantly depleted in nucleosome tags at the 5 % confidence level as compared to flanking windows; (G2) NIEBs significantly enriched in nucleosome tags at the 5 % confidence level as compared to flanking windows; and (G3) the remaining NIEBs. For “Valouev” in vitro data, we found that 76.7 % of the NIEBs are in G1 and correspond to regions where the experimental in vitro nucleosome tags density is in agreement with the NIEB predictions, and only 0.2 % are in G2 and have a significant nucleosome occupancy signal over the predicted NIEBs (Additional file 1: Figure S3A). Interestingly, similar distributions over the three groups were obtained when analyzing “Valouev” in vivo data (Additional file 1: Figure S3B), and also “Schones” in vivo data (Additional file 1: Figure S3C). Despite a significantly weaker sequencing depth in “Schones” in vivo data, we obtained similar percentages of NIEBs in G1 for the former (54.0 %) than for the latter (58.7 %), confirming that a majority of NIEBs correspond also to NDRs in vivo. Note that the corresponding percentages of NIEBs in (G2), namely 0.5 % in “Valouev” data and 0.9 % in “Schones” data, are both very small, as previously observed with in vitro data. Thus, independently of the initial strategy used to identify these regions of interest, the important point is that these AT-rich regions impair nucleosome formation in vitro and in vivo.

### In vivo nucleosome ordering near NIEBs is encoded in the DNA sequence

As expected from statistical positioning [19, 30], these NIEBs are flanked by a few (∼ 2-3) rather well-positioned nucleosomes with a nucleosome repeat-length (NRL) ∼160 bp robustly observed in vivo and slightly more pronounced in vitro (Fig. 1a, a’). This nucleosome ordering is in remarkable agreement with the normalized mean nucleosome density profiles predicted by the sequence-dependent physical model at low and high genome nucleosome coverages (Methods) with a robust NRL ∼150 bp (Fig. 1b, b’). This indicates that not only the NDRs but the neighbouring nucleosome arrangement are programmed in the DNA sequence. Indeed, the local GC content (Fig. 1c, c’) provides an excellent prediction of the experimental mean nucleosome occupancy (i.e. the probability that a given locus belongs to a DNA fragment involved in the nucleosome complex) profiles and this not only for in vitro data, as previously pointed out in various organisms, including *S. cerevisiae* [19, 53, 54, 58], *C. elegans* [19, 51, 53, 58] and human [41, 42], but also for in vivo data. Let us point out that a quite similar mean GC content profile is obtained when considering regions of the chimpanzee genome homologous to the human intrinsic NDRs (data not shown, Methods) suggesting that the nucleosome ordering associated with NIEBs is robust across primate evolution. Note that the intrinsic mean nucleosome spacing predicted by the physical model (Fig. 1b, b’) likely encoded in the local GC-content (Fig. 1c, c’) and consistently observed in vitro and in vivo (Fig. 1a, a’), namely NRL ∼150−160 bp, is significantly smaller than the in vivo genome average NRL ∼203 bp, the average heterochromatin NRL $\gtrsim 205$ bp and more surprisingly the average NRL ∼178 bp in euchromatin around active promoters and enhancers estimated in CD 4^+^ [42]. The highly compacted nucleosome arrangement predicted and observed on both sides of the NIEBs with a rather short DNA linker size ∼10−20 bp, is likely to affect and probably to impair the condensation of the nucleosomal array into the 30 nm chromatin fiber [59–63].

We reproduced this analysis of nucleosome occupancy nearby NIEBs according to the G1, G2 or G3 group classification. For each set of experimental data, taking into account the mappability of the considererd regions (Additional file 1: Figure S4), we plotted the mean nucleosome density (Additional file 1: Figure S5) and the mean GC content profile (Additional file 1: Figure S6). Whatever the group, the GC profile displays a robust oscillatory pattern similar to the one previously observed on the genome-wide mean GC profile (Fig. 1c, c’). As expected, a compact nucleosome arrangement like the one observed genome-wide (Fig. 1a, a’) was found around the G1 energy barriers that indeed constitute a large majority of predicted NDRs in the “Valouev” in vitro and also in the “Valouev” and “Schones” in vivo data. More importantly, when averaging the nucleosome tag densities around the set of 43,364 NIEBs lacking sufficient mappability to perform the test (Additional file 1: Figure S4), we recovered a consistent well ordered but less pronounced nucleosome ordering flanking the predicted NDRs in the in vitro and also in the two in vivo nucleosome data sets (Additional file 1: Figure S5). The additional observation that no significant departure from the compact nucleosome ordering picture was observed around the G3 energy barriers resulted in the following overall estimates: among the 1,581,256 NIEBs, 1,578,827 (99.8 %) in “Valouev” in vitro data, 1,573,887 (99.5 %) in “Valouev” in vivo data and 1,566,659 (99.1 %) in “Schones” in vivo data, correspond to experimentally observed NDRs bordered by rather well-positioned and strongly compacted neighbouring nucleosomes. Thus, only an extreme minority (<1 %) of the predicted NIEBs, namely the ones that belong to the group G2, were found to have a nucleosome occupancy higher than their two flanking 300 bp windows.

### NIEBs and intrinsic flanking nucleosomes are widely distributed over human autosomes

**Fig. 2:**
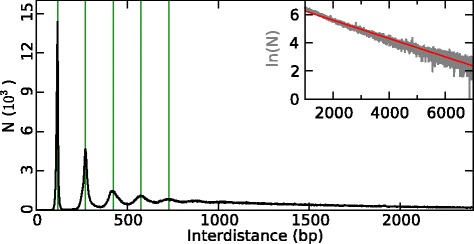
Histogram of (1,581,005) border to border inter-distances between successive NIEBs. The inset corresponds to a log-lin representation of the tail of this histogram, which puts into light a Poisson-like exponential behavior with a mean interdistance $\bar d=1,541$ bp (*red straight line*). The *green vertical lines* mark interdistances *d*=117+153 *k* (bp), *k*=0,1,2,3 and 4

**Fig. 3:**
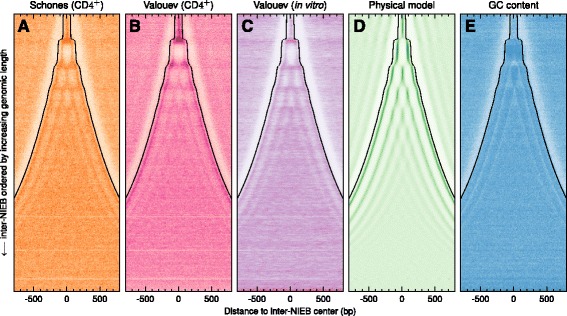
Heat map of nucleosome density and GC content in between NIEBs. The 1,581,005 inter-NIEB regions were centered at 0 and ordered vertically from the smallest (*top*) to the largest (*bottom*). **a** “Schones” in vivo data; (**b**) “Valouev” in vivo data; (**c**) “Valouev” in vitro data; (**d**) in silico nucleosome density computed at high nucleosome coverage (Methods); (**e**) GC content. Only the central part of inter-NIEB regions larger than 1.6 kb were shown to enlighten the absence of nucleosome positioning in these regions. Each horizontal line represents the mean nucleosome coverage over 200 inter-NIEB regions coded from white (0) to full color (1) (Methods)

To characterize the spatial distribution of these NIEBs along human chromosomes, we performed a statistical analysis of the border to border interdistances between successive NIEBs. The obtained histogram (Fig. 2) displays an exponential tail with a characteric interdistance compatible with the mean interdistance $\bar d=1.54$ kb as the signature of a Poisson-like distribution. Interestingly, for interdistances *d*<1 kb, this histogram switches to a quantized distribution with peaks equally separated by a remarkably stable and robust distance 153 bp, quite similar to the characteristic DNA length 147 bp involved in the nucleosome complex. We generated two-dimensional (2D) maps of nucleosome density that reveal a strikingly organized nucleosome ordering along inter-NIEB regions (Fig. 3). A crystal-like nucleosome organization with a well-defined number of regularly spaced nucleosomes is clearly observed in both “Schones” (Fig. 3a) and “Valouev” (Fig. 3b) in vivo data. As the interdistance *d* increases, the 1,581,005 inter-NIEB regions clusters into classes displaying the same number *n* of nucleosomes, from *n*=1 (145,463; 9.2 %), 2 (147,922; 9.4 %), 3 (117,678; 7.4 %), 4 (116,399; 7.4 %), to 5 (108,324; 6.9 %) for *d*<0.8 kb. For larger *d*, the periodic nucleosome positioning remains visible, but appears fuzzy as the signature of some weakening of the statistical positioning away enough from the two confining bordering NIEBs [19, 30, 32]. Nevertheless, a close inspection of the in vivo experimental 2D maps shows that if the central nucleosomes have no preferential positioning, the first two nucleosomes flanking the NIEBs are well positioned and highly compacted with a very short NRL ≃153 bp and this even for the largest inter-NIEB regions. This remarkable crystal-like nucleosomal chromatin architecture reminds us of the intragenic nucleosome ordering previously observed in *S. cerevisiae* [32], except that contrary to its vanishing in in vitro data in yeast, this periodic ordering is still observed in “Valouev” in vitro data in human (Fig. 3c) and in the physical model at low nucleosome coverage (Fig. 1b, b’) as the footprint of some underlying positioning sequences. This conclusion is corroborated by the quite similar 2D maps predicted by our physical model at low (in vitro) and high (in vivo) nucleosome coverages (Methods) that remarkably reproduce (Fig. 3d) the nucleosomal patterns observed in vivo (Fig. 3a, b) and in vitro (Fig. 3c). Indeed, when representing the corresponding heat map obtained for the GC content (Fig. 3e), we confirmed that nucleosome occupancy nearby NIEBs is intrinsically encoded in the local GC content, in the crystal-like nucleosome organization observed in small (*d*<0.8 kb) inter-NIEB regions, as well as in the first two flanking nucleosomes for larger (*d*>0.8 kb) inter-NIEB regions. Altogether, relative to the overall 2,681,301,120 bp of sequenced DNA in the 22 human autosomes, the 1,581,256 NIEBs cover 241,129,337 bp (9.0 %), the 635,786 inter-NIEB regions with crystal-like nucleosome ordering (*n*=1 to 5) cover 254,831,369 (9.5 %) and the well-positioned first two nucleosomes flanking the 945,219 widely spaced (*d*>800 bp) successive NIEBs cover 510,418,260 bp (19.0 %). This means that in 37.5 % of the human genome, the GC content provides a remarkable prediction of nucleosome occupancy. Thus, in more than a third of the human genome, in vivo nucleosome positioning is intrinsically encoded in the DNA sequence without evidence of local chromatin remodeling. These findings also suggest that nucleosome positioning could be at the basis of local GC content variation as opposed to a mutational basis such as bias gene conversion [64–66].

With regard to the large scale organization of mammalian genomes [4, 67], we investigated the density of NIEBs in 100 kb non-overlapping windows that we further classified according to their GC content, mean replication timing (MRT) data, DNase I sensitivity raw tag density and meiotic recombination rate respectively (Table 1). We used MRT [68] and DNase I sensitivity [69] data from the lymphoblastoid cell line Gm06990 as surrogates of corresponding data in CD 4^+^ (Methods). As compared to the genome-wide mean density =0.59 NIEB/kb, the densities obtained in the GC-poor light isochore regions L1 (0.54 NIEB/kb) and L2 (0.63 NIEB/kb) are not so different from the ones obtaines in the GC-rich heavy isochore regions H1 (0.65 NIEB/kb), H2 (0.56 NIEB/kb) and H3 (0.44 NIEB/kb) with a significant enrichment in L2 and H1 relative to some significant depletion in L1 and H3 (*P*≪10^−3^, Table 1). This non-monotonous variation of the NIEB density versus the GC content contrasts with the anticorrelation observed with the MRT, the early replicating windows containing relatively more NIEBs (0.65 NIEB/kb) than mid S phase windows (0.59 NIEB/kb) and late replicating windows (0.54 NIEB/kb) (*P*≪10^−3^, Table 1). Consistently with previous observation that early replicating regions are more sensitive to DNase I cleavage than late replicating regions [70, 71], we found that the NIEB density indeed correlates with DNase I tag density, the less sensitive 100 kb windows containing relatively less NIEBs (0.53 NIEB/kb) than the most sensitive (0.63 NIEB/kb) (*P*≪10^−3^, Table 1). No significant correlation was observed with the meiotic recombination rate with only a slight enrichment (0.61 NIEB/kb) in windows with low cross-over rate (Table 1). Since GC-rich, early replicating open chromatin regions accessible to DNase I digestion are known to be highly genic regions [72], we further investigated the NIEB density in genic and intergenic regions. Human genes contain slightly more NIEBs (0.61 NIEB/kb) than intergenic regions (0.57 NIEB/kb) (*P*≪10^−3^, Table 1). But as observed genome-wide, the density of NIEBs in the intergenic regions displays quite comparable anti-correlation with MRT and correlation with DNase I tag density (Additional file 1: Table S1), indicating that these trends do not simply reflect the difference in gene density in early replicating transcriptionally active open chromatin-regions relative to late replicating gene desert heterochromatin regions. Interestingly, as compared to genome average, the more (resp. less) accessible euchromatin (resp. heterochromatin) regions were found to be enriched (resp. depleted) in crystal-like nucleosomal patterns bordered by two NIEBs and this in genic as well as in intergenic regions (Additional file 1: Table S2). Fuzzy nucleosome occupancy in larger (*d*>0.8 kb) inter-NIEB regions were more homogeneously observed along human autosomes without significant correlation with GC content, MRT, DNase I tag density and meiotic recombination rate (Additional file 1: Table S2). Altogether these results show that the predicted 1,581,256 NIEBs are widely distributed along human autosomes, with a density at 100 kb resolution that robustly ranges from 0.54 to 0.65 NIEB/kb, meaning that on average, one can expect to find a NIEB every 1.5−1.8 kb along human chromosomes and this whatever the genomic (nucleotide composition, gene, intergene) and epigenetic (MRT, DNase I sensitivity) context. When adding to these NIEBs, the flanking nucleosomes that were also shown to be encoded in the local GC content (Fig. 3), this is indeed a ∼1 kb long constitutive nucleosomomal chromatin pattern that is widely distributed along human chromosomes covering overall more than a third of the genome.

**Table 1:** Large-scale distribution of the predicted 1,581,256 NIEBs along the 22 human autosomes

	(% genome)	NIEB/kb	cov (%)
All NIEBs	100	0.59	9.0
L1 (GC <38 %)	33.3	0.54	9.5
L2 (38≤ GC <42 %)	32.3	0.63	9.6
H1 (42≤ GC <47 %)	21.4	0.65	9.0
H2 (47≤ GC <52 %)	9.2	0.56	6.9
H3 (52 % ≤ GC)	3.7	0.44	5.0
Early (MRT <0.36)	30.0	0.65	9.1
Medium (0.36≤ MRT <0.69)	40.0	0.59	8.9
Late (0.69≤MRT)	30.0	0.54	9.1
Low DNase (<14.3 reads/kb)	30.0	0.53	9.0
Medium DNase	40.0	0.60	9.3
High DNase (>29.3 reads/kb)	30.0	0.63	8.7
Low Recomb (<0.378 cM/Mb)	30.0	0.61	9.7
Medium Recomb	40.0	0.59	9.0
High Recomb (>1.681 cM/Mb)	30.0	0.58	8.4
Genes	47.1	0.61	9.2
Intergenes	51.6	0.57	8.9

NIEB density was computed in 100 kb non-overlapping windows that were classified according to their GC content, MRT, DNase I sensitivity raw tag density and meiotic recombination rate (Methods). The last two rows correspond to mean NIEB densities computed in 23,329 genes (Methods) and complementary intergenic regions (Methods). The first column defines the region, the second column its corresponding genome coverage (%), the third column the mean NIEB density (NIEB/kb) and the fourth column, the corresponding coverage of this region by the set of NIEBs (%). For each row, we estimated the standard error of the mean NIEB density to be SEM <0.01

### Complex patterns of divergence around NIEBs

**Fig. 4:**
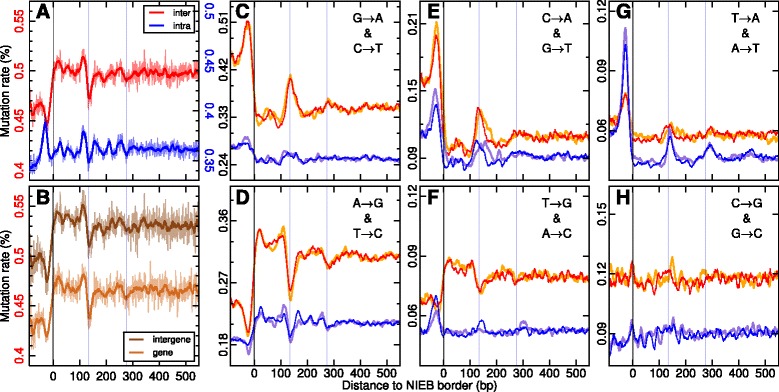
Mean human lineage-specific inter- (*red*, *orange*, *brown*) and intraspecies (*blue*, *purple*) divergence rates inside (distance to NIEB border <0) and nearby (distance to NIEB border >0) the 1,581,256 NIEBs. **a** Overall divergence rates; rate of interspecies (resp. intraspecies) divergence is plotted on the left-hand (resp. right-hand) y-axis. **b** Interspecies divergence rate in gene (*light brown*) and intergene (*dark brown*) regions. **c** G → A (*red*, *blue*) and C → T (*orange*, *purple*) substitution rates. **d** A → G (*red*, *blue*) and T → C (*orange*, *purple*) substitution rates. **e** C → A (*red*, *blue*) and G → T (*orange*, *purple*) substitution rates. **f** T → G (*red*, *blue*) and A → C (*orange*, *purple*) substitution rates. **g** T → A (*red*, *blue*) and A → T (*orange*, *purple*) substitution rates. **h** C → G (*red*, *blue*) and G → C (*orange*, *purple*) substitution rates. The *vertical blue lines* have the same meaning as in Fig. 1 a’–c’. In (**c**–**h**), the substitution rate on the right of the 3’ NIEB borders was averaged with its reverse complement on the left of the 5’ NIEB borders. Curves were smoothed over 10 bp windows. Non-smoothed curves are shown as a background in (**a**) and (**b**)

To detect possible evidence of selection linked to the nucleosomal organization of the chromatin fiber, we first used single nucleotide substitution rates tabulated in the human lineage since its divergence from chimpanzee using macaque and orangutan as outgroups [6] (Methods). We found that with respect to the mean substitution rate (0.50 %) obtained when averaging over the 22 human autosomes, the level of divergence in the NIEBs is significantly lower (0.46 %) (*P*≪10^−3^, Fig. 4a), revealing that these regions unfavourable to nucleosome formation were highly conserved, at least during recent evolution. As previously observed in yeast [9–13], medaka fish [15] and human [16–18], we recovered that the overall levels of divergence are higher than genome average in the well-positioned nucleosomes flanking the NIEBs and lower in linker regions but not as low as in the NIEBs. Interestingly, quite similar substitution rate patterns were obtained in genes and in intergenic regions (Fig. 4b), around a mean substitution rate that is larger (0.53 %) in intergenic regions than in genes (0.47 %) (*P*≪10^−3^), consistent with previous reports [5, 6] that higher substitution frequency is generally observed in late replicating, gene poor, heterochromatin regions. We computed the GC compositional profile toward which the sequences are evolving as the result of the so-computed substitution rate matrix (Methods). The resulting profile of GC composition at equilibrium (Fig. 1c, c’) is in remarkable agreement with the “Valouev” in vitro and “Valouev” and “Schones” in vivo experimental nucleosome occupancy data (Fig. 1a, a’), with a NRL ∼155-160 bp. This further supports the interpretation of local GC variation as a physical basis accommodating for a specific nucleosomal positioning pattern. When comparing this equilibrium GC profile to the GC profiles obtained from the native and repeat-masked sequences, some additional oscillations are present in the core of the flanking nucleosomes in the native GC profile, suggesting the presence nearby the NIEBs of sequences that do not reflect long-term evolutionary compositional patterns, e.g. repeated sequences that have been inserted recently in the human genome.

Next we examined the relative contributions of individual base changes. Strong peaks of S (C/G) → W (A/T) substitution rates were observed in the NIEBs (Fig. 4c, e) with matching low rates of W → S substitution rates in the same regions (Fig. 4d, f). As a consequence, there is a clear preference for substitutions increasing the AT content of these NIEBs. A similar preference was observed in the linker regions between the successive flanking nucleosomes but this preference progressively vanishes when moving away from the NIEBs (Fig. 4c–f). Different substitution patterns were obtained at the well-positioned flanking nucleosomes with high rates of W → S substitutions (Fig. 4d, f) coming along with low rates of S → W substitutions (Fig. 4c, e). This combination of divergence patterns contributes to increasing the GC content at the flanking nucleosome positions, consistent with previous analysis of well-positioned nucleosome in “Schones” in vivo data by Prendergast and Semple [18]. Note that its importance progressively damps out when considering nucleosomes further away from the NIEBs as expected from statistical ordering near an excluding energy barrier [19, 30].

Using single nucleotide polymorphism (SNP) data from several fully sequenced human genomes [73], we further investigated the rates of intraspecies divergence (Methods). The broad variation in total polymorphism density in and around the set of NIEBs is somehow different from the overall pattern of interspecies divergence (Fig. 4a). Among the dominating intraspecies divergence rates, we recovered in the recent human lineage strong peaks of S → W substitution rates in the NIEBs and to a lesser extent in the linker regions (Fig. 4c, e) that correlate with low rates of W → S substitutions in the same regions (Fig. 4d). Also both interspecies and intraspecies T → A and A → T substitution rates display some increase in the NIEBs (Fig. 4g). As far as the well-positioned flanking nucleosomes are concerned, we consistently found high rates of A → G and T → C intraspecies substitutions (Fig. 4d) with matching lower C → A and G → T substitution rates (Fig. 4e). But the comparison of inter- and intraspecies divergence rates also revealed striking differences for certain classes of substitutions. In particular the T → G and A → C intraspecies substitution rate profiles were found rather flat over the flanking nucleosome regions with some relative increase (and not decrease) in the NIEBs (Fig. 4f). These observed differences in rates of interspecies and intraspecies divergence strongly suggest the presence of some underlying modes of selection associated with nucleosome ordering nearby the NIEBs.

### The genomic sequences associated with NIEBs and flanking nucleosomes are not evolving neutrally

To investigate the possible existence of selection in the regions of interest, we used McDonald-Kreitman (MK) test and its variants [74] which amounts to compare rates of fixed interspecies divergence and intraspecies polymorphisms. Positive (resp. negative) selection is expected to lead to an excess (resp. depletion) of interspecies divergence relative to intraspecies divergence. To avoid to be biased by confounding factors like altered rates of mutation and repair [18] that potentially can induce differences in substitution rates in some regions, we not only computed the rates of inter- and intraspecies divergence at the sites of interest but also the rates of inter- and intraspecies divergence at distant loci with no preferential nucleosome positioning as a “proxy” of the rate of neutral divergence (Methods). Practically, we used the average rates of inter- and intraspecies divergence computed in the central regions of the 945,219 largest (*d*>0.8 kb) inter-NIEB regions, where nucleosome density was predicted (Fig. 3d) and observed experimentally (Fig. 3a–c), to be flat, as reflecting the background mutation rates (Methods). Thus, if the NIEBs and flanking nucleosomes were evolving neutrally, we would expect that the ratio of interspecies and intraspecies base changes corrected for “proxy” neutral rates, be robustly $S_{\mathcal {X} \to Y}=1$ in the regions of interest as further away from the NIEBs. If instead these regions were under selective pressure, we would expect to see some deviation $S_{\mathcal {X} \to Y}>1$ (positive selection) or $S_{\mathcal {X} \to Y}<1$ (negative selection) across the ∼1 kb regions encompassing the NIEBs and the flanking (on the right and on the left) well-positioned nucleosomes.

**Fig. 5:**
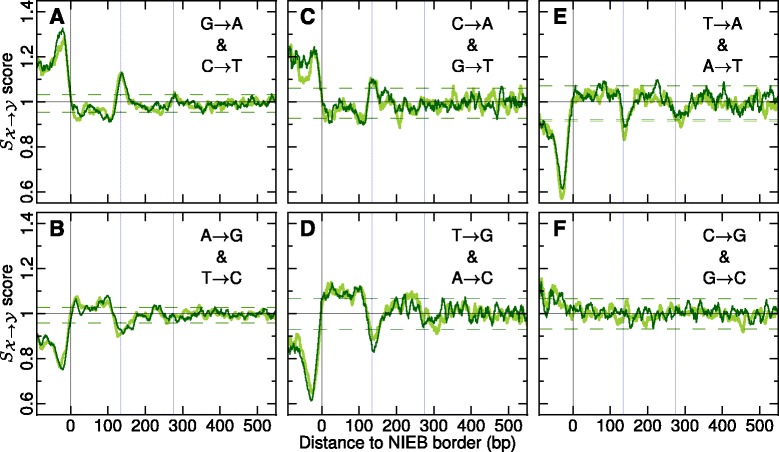
Evidence of selection in and around the 1,581,256 NIEBs. Ratios $S_{\mathcal {X} \to \mathcal {Y}}$ of background corrected inter- and intraspecies divergence rates plotted against the position from the closest NIEB border (negative distances correspond to loci inside the NIEBs). The pannels correspond to the substitution rates: (**a**) G → A and C → T; (**b**) A → G and T → C; (**c**) C → A and G → T; (**d**) T → G and A → C; (**e**) T → A and A → T; (**f**) C → G and G → C. In each panel the first (resp. second) substitution is represented in *dark green* (resp. light green). Curves were smoothed over 10 bp windows. The *vertical blue lines* have the same meaning as in Fig. 1 a’–c’. The *horizontal dark green* (resp. light green) dashed lines, mark the 95 % confidence intervals for $S_{\mathcal {X} \to \mathcal {Y}}$ ratios under the hypothesis of neutral evolution (Methods); the probability for $S_{\mathcal {X} \to \mathcal {Y}}$ to be above (resp. below) these limits is 0.025

As shown in Fig. 5, we obtained convincing signatures of selection in all W → S and S → W substitutions, but the observed patterns of selection look rather complex, with opposite forces of selection in the NIEBs and linkers regions, as compared to the flanking nucleosomes. For instance, a relative excess of the rates of S → W interspecies divergence compared to intraspecies divergence rates was found in the NIEBs and to a lesser extent in the linker regions as a indication of positive selection (Fig. 5a, c). The maximal $S_{\mathcal {X} \to Y}$ value observed over 10000 bootstrap simulations under the hypothesis of neutral evolution (Methods) is 1.07 (resp. 1.12) for G → A and C → T (resp. C → A and G → T) (Additional file 1: Figure S7). $S_{\mathcal {X} \to Y}$ values in NIEBs and linker regions are larger that these maximal values underlining the high significance (*P*≪10^−4^) of the positive selection acting on these regions. On the contrary, in the flanking nucleosomes some depletion of the same S → W interspecies substitution relative to intraspecies divergence rates was observed as indicative of purifying selection that progressively vanishes when considering nucleosomes further away from the NIEBs (*P*≲0.025 on the first nucleosome) (Fig. 5a, c). Interestingly, the opposite patterns of selection were found for W → S substitutions. Negative selection was observed in the NIEBs (*P*≪10^−4^) and linker regions (*P*<0.025) and positive selection in the neighbouring nucleosome positions (*P*<0.025 on the first nucleosome) (Fig. 5b, d). These individual complex selection patterns actually cooperate to the overall maintenance of lower GC composition in NIEBs and linker regions and of high GC composition in the flanking nucleosomes. Note that the selection patterns found around the dyads of the 5,474,320 well-positioned first two flanking nucleosomes in the present study are in perfect agreement with those previously reported by Prendergast and Semple [18] from a similar analysis of 817,774 well-positioned nucleosomes identified in “Schones” in vivo nucleosome occupancy data of which ∼30 % are located within 300 bp of a NIEB border.

**Fig. 6:**
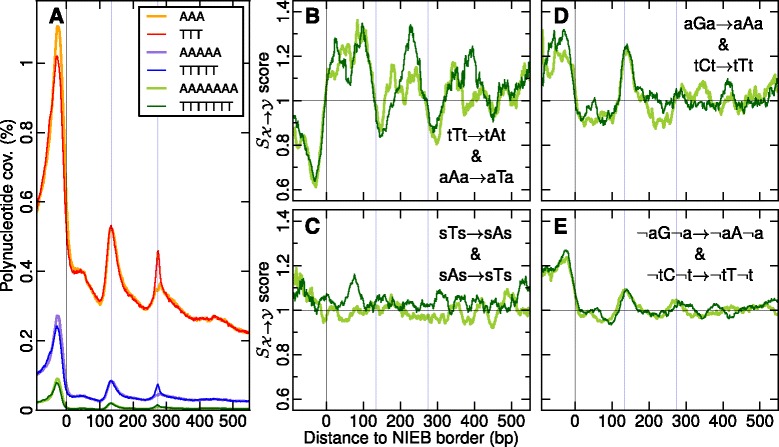
**a** Mean profile of (repeat-masked) polynucleotide coverage in and around the 1,581,256 NIEBs: AAA (*orange*), TTT (*red*), AAAAA (*purple*), TTTTT (*blue*), AAAAAAA (*light green*), TTTTTTT (*dark green*). Ratios $S_{\mathcal {X} \to \mathcal {Y}}$ of background corrected inter- and intraspecies context-dependent divergence rates plotted against the position from the closest NIEB border. The pannels correspond to the substitution rates: (**b**) tTt → tAt and aAa → aTa; (**c**) sTs → sAs and sAs → sTs, where s =(c,g); (**d**) aGa → aAa and tCt → tTt; (**e**) ¬a*G*¬a →¬a*A*¬a and ¬t*C*¬t →¬t*T*¬t, where ¬*a*=(c,t,g) and ¬*t*=(a,c,g). In each panel the first (resp. second) substitution is represented in *dark green* (resp. light green). In (**b**, **c**, **d**, **e**) curves were smoothed over 30 bp windows. The *vertical blue lines* have the same meaning as in Fig. 1 a’–c’

As compared to the rather flat selection score profile $S_{\mathcal {X} \to Y} \simeq 1$ indicating the absence of selection in C → G and G → C substitutions in the flanking nucleosomes and a very weak positive selection in the NIEBs (Fig. 5f), some signature of purifying negative selection was observed in T → A and A → T in the NIEBs (*P*≪10^−4^) and in the linkers (*P*<0.025) (Fig. 5e). This reflects the presence of highly conserved poly(A) and poly(T) patterns (Fig. 6a) that are known to impair nucleosome formation [43, 55, 58] and favour nucleosome disassembly [52] by increasing the DNA wrapping free energy cost around the histone octamer. Interestingly, besides being globally AT-rich, the NIEBs are delimited on each side by these rigid DNA fragments that indeed contribute to delineate their edges. The additional estimation of context-dependent substitution rates proved that tTt → tAt and aAa → aTa substitutions are highly anti-selected (Fig. 6b) on the opposite to sTs → sAs and sAs → sTs (Fig. 6c), where s =(c,g), confirming that these poly(dA:dT) patterns are evolutionary conserved. Besides this negative selection to disrupt poly(A) and poly(T), we also found some positive selection in aGa → aAa and tCt → tTt (Fig. 6d) and also in aCa → aAa and tGt → tTt (data not shown) to create these nucleosome inhibitory patterns at the NIEB edges and in the linkers, that slightly exceeds the one observed in ¬a*G*¬a →¬a*A*¬a and ¬t*C*¬t →¬t*T*¬t (Fig. 6e), where ¬*a*=(c,t,g) and ¬*t*=(a,c,g). These results provide some clear understanding of the localized excess of positive selection in S → W substitutions at the NIEB edges and in the linker (Fig. 5a, c) concomitant with a localized excess of negative selection in W → S substitutions (Fig. 5b, d).

To avoid possible biais in the MK test-inspired analysis induced by spurious low frequency polymorphisms [75], we took into account in our study only SNPs with a minor allele frequency >1 %, which represent 15 % of the original sampling set only. However, when considering every SNP, we recovered very similar complex selection patterns with only little differences (Additional file 1: Figure S8), confirming that these signatures of positive and negative selections were maintained even in presence of slightly deleterious mutations.

As a control study, we reproduced our analysis of inter- and intraspecies substitution rates after removing repeated sequences using RepeatMasker (Methods). This had little effect on the broad selection patterns previously observed (Additional file 1: Figure S9). Note that our demonstration is based on the observation that similar oscillating mean GC content profiles are found in chimpanzee and human nearby homologous intrinsic NDRs suggesting a robust nucleosome ordering in these regions (no nucleosome repositioning [76]) across primate evolution.

### Selection maintains optimal GC profile for nucleosome positioning nearby NIEBs

**Fig. 7:**
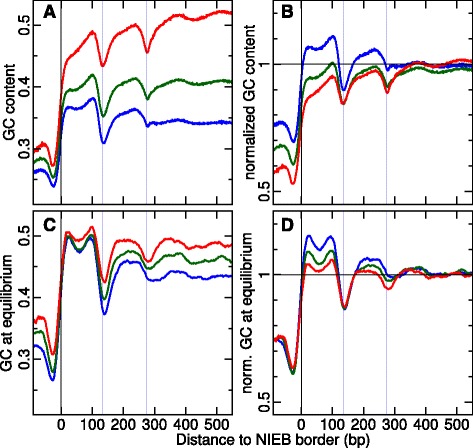
**a** Repeat-masked GC content in and around the predicted NIEBs bordering the 945,219 largest (*d*>0.8 kb) inter-NIEB regions. These barriers were classified according to the repeat-masked GC content computed in the central part of these large inter-NIEB regions: GC <0.38 (*blue*), 0.38≤ GC <0.46 (*green*) and 0.46≤ GC (*red*). **b** Repeat-masked GC content normalized by its local repeat-masked reference value. **c** Same as (**a**) for GC content at equilibrium. **d** Equilibrium GC content normalized by its local equilibrium reference value. In (**c**, **d**) curves were smoothed over 30 bp windows. The *vertical blue lines* have the same meaning as in Fig. 1 a’–c’

Even though NIEBs were shown to be rather homogeneously distributed in GC-rich and GC-poor isochores (Table 1), one may wonder whether the local GC composition has some effect on the divergence rates observed in the NIEBs as well as in the flanking nucleosomes and linker regions. As shown in Fig. 7a, b, when using as a reference the average GC content computed in the central regions of the 945,219 largest (*d*>0.8 kb) inter-NIEB regions, we clearly evidenced significant differences in the GC content profiles obtained for different classes of local reference GC content. The higher the local GC content, the largest the relative GC composition depletion inside NIEBs and linker regions, and in contrast the lowest the relative GC composition excess at the flanking nucleosome positions. In particular, for local GC ≥46 %, the GC composition inside the flanking well-positioned nucleosomes shows almost no difference with the reference GC content characteristics of background ill-positioned nucleosomes. The computation of the corresponding GC profiles at equilibrium (Methods) confirmed the existence of an optimal GC content at human nucleosome cores (Fig. 7c) consistent with previous study of mononucleotide and 5mer frequencies associated with the set of well-positioned nucleosomes identified in “Schones” in vivo data [18]. In contrast, the equilibrium GC content in NIEBs still depends on the reference GC content so that the difference in equilibrium GC content between NIEBs and inter-NIEB central regions is constant (Fig. 7d). Hence, the evolution of NIEB GC content is relative to its local GC environment.

To explicitely quantify how selection patterns were affected by the local GC content, we re-examined the modes and strengths of selection according to which class of reference GC content the considered inter-NIEB region belongs to (Additional File 1: Figure S10). We observed that the excess of W → S and in particular of A → G and T → C interspecies substitution rates compared to intraspecies rates at flanking nucleosome positions is stronger in a low GC environment and almost vanishing in a high GC environment (Additional file 1: Figure S10B,D). More significantly, the purifying selection observed at these flanking nucleosomes for S → W is stronger in low GC local environment and almost absent in high GC environment (Additional file 1: Figure S10A, C), as the signature of selection mainly acting to counterbalance the local high mutation rates towards high AT content. As far as the NIEBs and linker regions are concerned, no clear effect on the reference GC composition was observed on the positive and negative selection scores (Additional file 1: Figure S10) strongly suggesting the robustness of these selection patterns as regards to the local compositional environment. Altogether, these results confirm that the NIEBs and flanking nucleosomes correspond to chromatin patterns that have been selected during evolution according to complex selection patterns involving positive and purifying selection. The strengths and directions of selection depend on the local background GC content and combine to maintain a high difference in GC composition (Fig. 7c, d) between the lowest GC composition in the NIEBs and the highest GC composition in the closest flanking nucleosomes.

### NIEBs at gene transcription start and termination sites

Since the intrinsic chromatin pattern made of a NIEB and its flanking nucleosomes was found almost equally distributed in genes and in intergenic regions (Table 1, Additional file 1: Table S1), we repeated our analysis by focusing on the 790,285 totally intergenic chromatin patterns. Quite similar patterns of selection were obtained (Additional file 1: Figure S11) demonstrating that the different modes and strengths of selection evidenced in this study (Fig. 5) are generally acting over the entire human autosomes shaping the genome landscape not only relative to functional regulatory sequences, genes and exonic sequences submitted to coding selective pressure, but also in Mb long heterochromatin intergenic regions that were not known to be constrained to maintain adequate nucleosome organization. When focusing our analysis at a smaller scale around human gene TSS, we found some significant depletion of NIEBs coverage in a [–500 bp, 500 bp] window (Additional file 1: Figure S12A). Indeed as originally observed in vitro by Valouev et al. [42] (Additional file 1: Figure S13B), the sequence-dependent physical model confirmed a nucleosome positioning preference on these regulatory sequences and this for expressed as well as non-expressed genes (Additional file 1: Figure S13A) [77]. As compared to genome average density, a notable excess in nucleosome density is actually programmed to extend over a few kbs upstream of the TSS in the promoter region and also downstream in the gene body. This excess is predicted to be slightly more important for expressed than for non-expressed genes (Additional file 1: Figure S13A), in agreement with the in vitro data (Additional file 1: Figure S13B). These results are quite consistent with the emerging idea that in human, gene promoters, and more generally regulatory sequences, are basally protected by a high nucleosome occupancy [41]. When comparing the intrinsic (Additional file 1: Figure S13A) and in vitro (Additional file 1: Figure S13B) nucleosome density profiles around human gene TSS to the ones observed in vivo by Valouev et al. [42] (Additional file 1: Figure S13C) and Schones et al. [57] (Additional file 1: Figure S13D), we recovered for active genes, a situation previously observed in other organisms such as yeast and fly, with a NDR of ∼150 bp overlapping the TSS and flanked on both sides by an array of well-positioned and phased nucleosomes [77]. These NDRs and strong nucleosome ordering can be explained as resulting from the binding of Pol II acting as an “exluding” energy barrier that contributes to the statistical packing of neighbouring nucleosomes [42, 57]. This likely explains part of the ∼70 % of well-positioned nucleosomes found in “Schones” in vivo data away (>300 bp) from NIEB borders. This pronounced nucleosome depletion and nearby nucleosome positioning and phasing are actually lost in inactive promoters (Additional file 1: Figure S13C,D) that display rather flat nucleosome density profiles. This suggests that the transition from an inactive to an active promoter involves, in a Pol II-dependent manner, nucleosome sliding and/or nucleosome eviction [28, 42, 57]. Because genes regulation in higher eukaryotes is typically cell-type specific, it thus seems more advantageous to keep the promoters and related regulatory sites intrinsically occupied by nucleosomes unless they need to be used. Note that, as expected by the presence in most protein-coding genes of a poly(A) signal at their 3’ end, we observed a remarkable enrichment in NIEBs at TTS (Additional file 1: Figure S12B). As predicted by the sequence-dependent model (Additional file 1: Figure S13A’), and observed in vitro (Additional file 1: Figure S13B’), an intrinsic NDR is programmed at gene TTS with well-positioned flanking nucleosomes, a chromatin pattern which appears to be significantly remodeled in vivo (Additional file 1: Figure S13C’, D’).

### Mutational pattern at NIEBs cannot be explained by biased gene conversion

Several studies have identified biased gene conversion (BGC) as a neutral process leading to strong variation of W → S mutation rates along human chromosomes via the biased repair of A:C and G:T mismatch through meiotic recombination [64–66]. Thus one may wonder to which extent BGC may underly some of the base changes observed in our study. Here, we have identified large variations that affect all type of W → S and S → W substitution rates. These variations are not specific of GC-rich, nucleosome-poor regions of the human genome which are known to experience higher recombination rates [78]. We have shown that the selected chromatin patterns involving intrinsic NDRs and flanking nucleosomes are equally distributed in high, medium and low meiotic recombination rate regions (Table 1 and Additional file 1: Table S1). Moreover we found that, independently of their size, double strand break (DSB) hotspots in human individuals [79] are highly depleted in NIEBs (Additional file 1: Figure S14A, B) and consistently that the NIEBs are void of DSB hotspots (Additional file 1: Figure S14C). Altogether, these results demonstrate that the complex selection patterns revealed in this study, their dependence on the local GC content and the highly conserved AT-rich intrinsic NDRs cannot be explained by the neutral BGC process.

## Discussion

Nucleosome organization along the so-called 10 nm chromatin fiber definitely conditions the next steps of DNA compaction into successive higher order structures including condensation into the 30 nm fiber and the formation of chromatin loops, up to a full extent of condensation in metaphase chromosomes [3]. The existence of a (∼1 kb) chromatin pattern made of an intrinsic NDRs flanked by highly compacted and well-positioned nucleosomes (NRL ∼150-160 bp) encoded in the sequence and almost homogeneously Poisson-like-distributed along human chromosomes with a mean interdistance ∼1.5 kb that does not depend much on the GC content of the region, the gene density and the MRT, and that overall cover about 37.5 % of the genome, raises the question of which chromatin structure has been selected during the evolution and if so, to favour or facilitate which function? According to geometrical modelling of the constitutive 30 nm chromatin fiber [59–63], a small nucleosome spacing with a rather short DNA linker size ∼10-20 bp is likely to impair the condensation of the nucleosomal array into the chromatin fiber, leaving a well-organized chromatin structure open and accessible. But it is well documented that in somatic cells, there are regions of open accessible euchromatin, but also regions of highly repressive heterochromatin. We have reproduced our substitution and selection analysis after classifying our chromatin patterns according to the epigenetic chromatin state they belong to in the Gm12878 differentiated cell type. Following the multivariate analysis of epigenetic marks at 100 kb resolution [72, 80], we considered on the one hand chromatin patterns in gene rich, high GC, early constant replication timing regions (CTRs) enriched in open chromatin marks (e.g. H3K4me3, H3K27ac) and on the other hand chromatin patterns in gene desert, low GC, late CTRs enriched in the repressive heterochromatin mark H3K9me3 associated with the heterochromatin protein 1 (HP1). We found quite similar selection patterns as observed genome-wide (Fig. 5) for both classes of chromatin patterns (Additional file 1: Figure S15A–F), confirming that they likely operate uniformly and robustly along human chromosomes. These results suggest that an open and accessible basal 10 nm chromatin fiber has been selected in human to intrinsically facilitate the epigenetic regulation of nuclear functions in a cell-type-specific manner. This interpretation is strongly supported by the additional observation of quite similar selection patterns in pluripotent H1hesc cell line (Additional file 1: Figure S15A’-F’), which is known to have a highly dynamic and accessible chromatin refractory to both HP1 and polycomb heterochromatin spreading [72]. These results are consistent with the recent visualization at nanoscale resolution of the organization of the nucleosomes in intact nuclei and in single cells in human and mouse [81]. In pluripotent as well as in differentiated cell types, the reported experimental results argue against the existence of a highly ordered secondary structure such as the 30 nm chromatin fiber.

Repetitive mobile elements constitute nearly half of the human genome [67]. During this study, in multiple occasions, we have noticed that the results obtained with the native DNA sequence were slightly different from the ones obtained with the repeat-masked sequence, as examplified by the native and masked GC content profiles around the NIEBs (Fig. 1c, c’). As compared to the rather smooth two bump masked GC profile that perfectly matches in vitro and in vivo nucleosome occupancy data, the native GC profile displays some striking robust oscillatory internal patterns suggesting the presence of some repeated sequences nearby a non negligible subset of the predicted NIEBs. When systematically investigating the principal families of interdispersed repeats SINES (Alu, MIR) and LINES (Line1, Line2), we found that a lot of (52 %) Alu elements were inserted flanking a NIEB. The Alu retrotransposons have been inserting in primate genomes for the past 65 million years and have reached over one million copies covering ∼11 % of the human genome [82]. A typical dimeric Alu element is about 300 bp long and is composed of two distinc GC- and CpG-rich monomers, separated by a short A-rich region. Importantly, the 3’ end of an Alu element has a longer poly(A) track that plays a critical role in its amplification mechanism [82, 83]. Interestingly, we found that the orientation of the Alu element is strongly dependent on which NIEB side they were inserted. They are mainly sense at the NIEB 5’ end and antisense at the NIEB 3’ end (Additional file 1: Figure S16A), so that the body of the Alu element is external to the NIEB. The remarkable phasing of the Alu elements at NIEB 5’ end (resp. 3’ end) results from the matching of the poly(A) tail of the sense (resp. antisense) Alu with the poly(A) (resp. poly(T)) tracks that were shown to define the edges of some of the predicted NIEBs (Fig. 6a) leading to an asymmetric distribution of poly(A) and poly(T) tracks at the NIEB borders (Additional file 1: Figure S16B). However, a majority (61 %) of NIEBs are free on either side of Alu elements and do not result from mechanisms underlying Alu integration [82]. Indeed, for these Alu-free NIEBs, we observed a symmetric enrichment of poly(A) and poly(T) tracks at NIEB borders (Additional file 1: Figure S16C). Hence, NIEBs likely preexists Alu insertion and provide adequate chromatin patterns favorable to Alu integration. A systematic analysis of the spatial distribution of the Alus in relation to NIEBs and related chromatin patterns, should shed a new light on Alu integration and evolution as well as on their role in the chromatin-mediated regulation of gene expression and of the replication spatio-temporal program.

## Conclusions

Pioneering studies in yeast have shown that rates of divergence are higher in nucleosome cores than in linker DNA [9, 10]. Various interpretations of these fluctuations in divergence rates have been proposed, including the possible consequence of the limited access of DNA wrapped around the histones to DNA repair proteins [11] and/or the existence of purifying selection in linker regions to maintain correct positioning of nucleosomes [9, 12, 13]. AT-rich linker regions relative to GC-rich nucleosomal sequences were further shown to evolve under compensatory dynamics that maintain heterogenous level of AT content through spatially coupled W(A/T)-losing and W-gaining substitutions [48]. The recent analysis of well-positioned nucleosomes in CD 4^+^ human T-cells [18] has revealed the existence of similar substitution patterns in the linker regions as a part of a complex combination of positive and negative selection acting to maintain a favourable base compositions in both linker and core regions of human nucleosomes. But in regard to the nucleosomal organization of the chromatin fiber, these signatures of selection were obtained from a relatively small subset of well-positioned nucleosomes that cover less than 5 % of the human genome [18]. In this study, we have developed a similar analysis of modes of selection associated with nucleosome occupancy along human chromosomes, taking as reference chromatin sequences no longer the ones favouring nucleosome positioning but instead the ones hindering nucleosome formation as predicted by a physical model based on sequence-dependent DNA bending properties. We have shown that these 1,581,256 NIEBs of mean size 153 bp together with their first two flanking nucleosomes are actually encoded in the GC content and are widely distributed along the 22 human autosomes in GC-rich and GC-poor isochores, in early and late replicating regions, in intergenic and genic regions (Table 1). By comparing rates of inter- and intraspecies divergences, we have brought evidence of complex patterns of positive and negative selection that depend on the local GC content to maintain some optimal difference in GC composition between the AT-rich intrinsic NDRs and the GC-rich well-positioned first flanking nucleosomes. This set of selected chromatin patterns (∼1 kb) made of well-positioned nucleosomes (∼ two on each side) flanking an intrinsic NDR overall cover about 37.5 % of the human genome, confirming that nucleosome positioning and antipositioning are likely to impact the divergence of many regions traditionally considered as evolving neutrally, such as intronic and intergenic DNA. We hope that the generalization of our study to other (non primate) mammalian and eukaryotic genomes will provide important clue to understanding genome evolution and epigenetic regulation in both health and disease.

## Methods

All this work was done on the 22 autosomes of the hg18 human assembly. For data coming from hg19 assembly, we used liftOver to convert coordinates from hg19 to hg18.

*Physical model*

We used the physical model based on sequence-dependent DNA bending properties described in [19, 34, 35]. For the prediction of NIEBs, we started with the set of 1,747,548 NIEBs detected over the 22 human autosomes by the physical model (see Section 3.4 in [77]), from which we discarded the largest ones (*l*>450 bp) that were shown to have non negligible in vivo nucleosome occupancy. All our analysis was based on the 1,581,256 resulting NIEBs. Note that when we studied the inter-NIEB regions (Fig. 3), we did not take into account the 229 non-fully sequenced inter-NIEBs. For the in silico nucleosome density, we used the same parameters than those detailed in Section 3.2 and 3.3 in [77] for modeling low (resp. high) nucleosome coverage observed in vitro (resp. in vivo). We used liftOver to determine the coordinates of the chimpanzee regions homologous to human NIEBs (liftOver from hg18 to panTro4).

*Genome-wide DNase I sensitivity data*

DNase I sensitivity raw tag density data from Gm06990 cells [69] were downloaded from the UCSC FTP site (http://hgdownload.cse.ucsc.edu/goldenPath/hg18/encodeDCC/wgEncodeUwDGF/) as a bed file (wgEncodeUwDGFSignalGm06990.bedGraph4.gz).

*Genome-wide nucleosome positioning data*

We used genome-wide maps of nucleosome positioning in vivo in resting human CD 4^+^ T cells and in vitro obtained by Valouev et al. [42] using micrococcal nuclease (MNase) digestion of chromatin and massive DNA sequencing technique (MNase-seq). Data files were downloaded from Gene Expression Omnibus (GEO, www.ncbi.nlm.nih.gov/geo/) under accession number GSE25133. For every read on the positive strand, we count a score of ‘1 read’ at the corresponding dyad locus, i.e. 70 bp further. We do the same for the negative reads, we count ‘1 read’ 70 bp before the read match.

We also used for comparison, the in vivo genome-wide map of nucleosome positioning in resting human CD 4^+^ T cells obtained by Schones et al. [57] using MNase-seq technique. We downloaded the tag coordinate bed files of the resting nucleosomes from dir.nhlbi.nih.gov/papers/lmi/epigenomes/hgtcellnucleosomes.aspx. As for “Valouev” nucleosome data, for every read ‘+’ (resp. ‘-’), we count a score of ‘1 read’ at the corresponding dyad locus, i.e. 70 bp further (resp. before).

For the heat maps of nucleosome density (Fig. 3a–c), we draw, for each NIEB, a horizontal segment around all loci where some tags were observed, of length equal to twice the number of tags, and then each line corresponds to the average over 200 NIEBs.

Both “Valouev” and “Schones” nucleosome data take into account only uniquely mapped reads (of length ∼25 bp). To avoid to be biased by the fact that some 25 bp sequences are repeated in the human genome, and therefore cannot be uniquely mapped, we considered, in our analysis, only sites with a mappability equal to 1 [84].

Schones et al. [57] well-positioned nuclesosome data as used in [18] were obtained from the weighted sequence model of Reynolds et al. [85].

*Statistical analysis*

To test whether the observed mean nucleosome depletion observed in the predicted set of NIEBs actually reflects MNase-seq profile across individual NIEBs, we compared nucleosome tag densities within the barriers and in the two 300 bp flanking windows (Additional file 1: Figure S3). To do so, we used a *Z*-test. For each NIEB, we computed the mean number *m*_1_ of tags per locus within the NIEB and the mean number *m*_2_ of tags per locus within the two 300 bp flanking windows as well as their associated standard deviation, *σ*_1_ and *σ*_2_ respectively. As explained above, we took into account only the *n*_1_ (resp. *n*_2_) mappable sites of these two regions. To perform the *Z*-test, *n*_1_ and *n*_2_ have to be >30. Thus, 43,364 NIEBs (2.7 % of our sample) were excluded from our analysis for lack of sufficient mappability. For the remaining 1,537,892 mappable NIEBs and flanking regions, we computed a *z*-score as:

1 $$  z = \frac{m_{2}-m_{1}}{\sqrt{\frac{{\sigma_{1}^{2}}}{n_{1}}+\frac{{\sigma_{2}^{2}}}{n_{2}}}} \; \;.  $$

Under the null hypothesis (there is as many tags inside NIEBs as besides them), this *Z*-test can be approximated by a normal distribution. Hence, there is 5 % of chance to get a *z*-score >1.645 (and 5 % of chance to get a *z*-score <−1.645). For each MNase-seq data set, we classified the 1,537,892 mappable NIEBs into 3 groups: (G1) NIEBs significantly depleted in nucleosome tags at the 5 % confidence level as compared to flanking windows (*z*-score >1.645); (G2) NIEBs significantly enriched in nucleosome tags at the 5 % confidence level as compared to flanking windows (*z*-score <−1.645); and (G3) the remaining NIEBs (−1.645≤*z*-score ≤1.645).

When comparing the mean value of two distributions (of GC content, DNase I HS site coverage, nucleosome coverage, NIEB density or substitution rate) obtained for two classes of region (NIEB *vs.* genome wide, NIEB *vs.* flanking regions, different classes of 100 kb windows, gene *vs.* intergene, …), we used the one-sided Welch’s unequal variance t-test, where the t-test distribution was approximated by a normal distribution.

*Interspecies divergence rates*

We used the point-mutation data obtained by Chen et al. [6]. Nucleotide substitutions were tabulated in the human lineage since its divergence from chimpanzee using both the orangutan and macaque as outgroups. To minimize the effects of alignment artifacts, only isolated substitutions defined as those flanked by sites that are identical in the four species were tabulated. Sequences were divided into CpG and non-CpG sites. CpG sites were defined as the sites having the following human/chimpanzee/orangutan/macaque pattern: NG/CG/CG/CG or CG/NG/CG/CG or CN/CG/CG/CG or CG/CN/CG/CG, where N is any nucleotide. CpG sites were excluded for the computation of C → W/S substitution rates. Substitution rates were calculated by dividing the number of substitution events of the appropriate type by the number of potentially mutable sites that meet the same criteria. Since the divergence between these four catarrhini species is small, possible multiple substitutions were ignored (using two outgroups instead of one lowers the amount of multiple mutations, in particular at CpG sites, and preferentially eliminates sites that are not ancestral to human and chimpanzee).

*GC composition at equilibrium*

We computed the GC profile towards which the sequences are evolving as the result of the observed substitution profiles without taking into account neighbor-dependency, including CpG substitutions.

*Intraspecies divergence rates*

We used the SNP data from The 1000 Genomes Project [73] (http://www.ncbi.nlm.nih.gov/variation/tools/1000genomes/). First, we dowloaded the ASN.1 flat files from ftp://ftp.ncbi.nlm.nih.gov/snp/organisms/human_9606_b142_GRCh37p13/ASN1_flat/ and we did a liftOver, from hg19 to hg18, of the SNP coordinates. Then, the ancestral base at any position was assumed to be any observed allele (including that observed in the reference genome) that matched the corresponding base in the ancestral genome (the one defined above for the definition of the interspecies divergence rates), or if this latter is not defined, the corresponding base in the chimp genome. Sites where the ancestral base could not be determined were excluded. As for interspecies rates, sequences were divided into CpG and non-CpG sites and CpG sites were excluded for the computation of C → W/S substitution rates.

Note that because MK test-inspired analyses were shown to be potentially skewed by the presence of slightly deleterious mutations (disproportionately segregate at low frequencies) [75], all this work was performed by taking into account only alleles observed in more than 1 % of the genomes, which represent only 15 % of the total amount of SNPs described in the files. However, this had little effect on the broad patterns seen in our analysis (Additional file 1: Figure S8 as compared to Fig. 5).

*Rates of selection*

The rate of base changes at each position from the NIEB border was measured by dividing the observed number of base changes by the total number of matching ancestral bases at each position [18]:

2 $$  dInter_{\mathcal{X} \to \mathcal{Y}} = \frac{InterDiff_{\mathcal{X} \to \mathcal{Y}}}{ancestralBase_{\mathcal{X}}} \; \;,  $$

3 $$  dIntra_{\mathcal{X} \to \mathcal{Y}} = \frac{IntraDiff_{\mathcal{X} \to \mathcal{Y}}}{ancestralBase_{\mathcal{X}}} \; \;.  $$

Equations 2 and 3 calculate the rates of base change at given positions from the NIEB border. $\mathcal {X}$ and $\mathcal {Y}$ correspond to the bases before and after the specific change, respectively, $\mathcal {X}$ being the ancestral base and $\mathcal {Y}$ being the base observed in the human lineage. $InterDiff_{\mathcal {X} \to \mathcal {Y}}$ and $IntraDiff_{\mathcal {X} \to \mathcal {Y}}$ are the total number of relevant interspecies and intraspecies changes observed at the position of interest relative to the NIEB border. $ancestralBase_{\mathcal {X}}$ is the number of corresponding ancestral bases observed at the same position.

Positions relative to the NIEB border where interspecies rates of change showed unusual deviations from intraspecies rates were identified by first correcting each value of $dInter_{\mathcal {X} \to \mathcal {Y}}$ and $dIntra_{\mathcal {X} \to \mathcal {Y}}$ for mean/background rates of divergence. Mean/background rates of change, *backgroundInter* and *backgroundIntra*, were estimated by averaging over the central positions in the middle (±300 bp) of the largest inter-NIEBs (*l*>800 bp). Those regions look like having rather flat inter- and intraspecies divergence rates (Fig. 4). (Note that for the Additional file 1: Figure S10, associated to the Fig. 7, three different backgrounds were computed for each base changes $\mathcal {X} \to \mathcal {Y}$: one for each class of local GC content.) The rate of interspecies base changes observed across all NIEBs and flanking regions at each position was then divided by the corresponding rate of intraspecies change to provide an indication of selection:

4 $$  S_{\mathcal{X} \to \mathcal{Y}} = \frac{dInter_{\mathcal{X} \to \mathcal{Y}}/backgroundInter_{\mathcal{X} \to \mathcal{Y}}}{dIntra_{\mathcal{X} \to \mathcal{Y}}/backgroundIntra_{\mathcal{X} \to \mathcal{Y}}} \; \;.  $$

The ratio of background corrected inter- and intraspecies divergence rates was calculated for each base change and at each position from the NIEB border. $S_{\mathcal {X} \to \mathcal {Y}} > 1$ indicates an excess of interspecies change and positive selection relative to mean rates, and values $S_{\mathcal {X} \to \mathcal {Y}}<1$ indicates an excess of intraspecies changes and negative selection.

In order to determine the $S_{\mathcal {X} \to \mathcal {Y}}$ values clearly distinguishing a hypothesis of selection from a hypothesis of neutral drift, we determined the expected $S_{\mathcal {X} \to \mathcal {Y}}$ distributions under the neutral hypothesis as the distributions obtained for the middle of the largest inter-NIEBs (*l*>800 bp). Given the 10 bp resolution adopted in this work, we randomly selected a 10 bp window in each of the large inter-NIEBs to compute one realization of $S_{\mathcal {X} \to \mathcal {Y}}$ values (Eq. (4)) under the neutral hypothesis. Repeating this operation 10000 times, we obtained the expected $S_{\mathcal {X} \to \mathcal {Y}}$ histograms (Additional file 1: Figure S7). We derived from these distributions 95 % confidence intervals for $S_{\mathcal {X} \to \mathcal {Y}}$ under the neutral hypothesis. Note that since large inter-NIEBs are only a fraction of the total number of regions averaged in Fig. 5 closed to and within the the NIEBs, these confidence intervals are conservative estimates. As expected, these intervals nicely bound the $S_{\mathcal {X} \to \mathcal {Y}}$ fluctuations away from the NIEBs (Fig. 5) illustrating that they can be used to visually assert the expected $S_{\mathcal {X} \to \mathcal {Y}}$ fluctuations without performing an explicit simulation in Fig. 6 and Additional file 1: Figures S8–S11 and S15). Note that since each 10 bp non-overlapping window along an observed *S* ratio curve (Fig. 5) is an independent observation of a *S* ratio value, it is justified to compute the *P*-value of these values using the expected $S_{\mathcal {X} \to \mathcal {Y}}$ histograms (Additional file 1: Figure S7) without correcting for multiple tests.

*Meiotic recombination*

Crossover rate data of the human genome were retrieved from the International HapMap Project (http://www.hapmap.org) [86]. The crossover rate for a given 100 kb window (Table 1) was computed by taking the two most distant markers belonging to this window and dividing their distance in the genetic map in centimorgans (CM) by their distance in the sequence map in megabases (Mb). Note that only the 100 kb windows with at least 5 markers were considered. We used the genome-wide recombination initiation maps of Pratto et al. [79] (Additional file 1: Figure S14). We downloaded the double strand break (DSB) hotspots in human individuals data (the GSE59836 peak data file) from http://www.ncbi.nlm.nih.gov/geo/query/acc.cgi?acc=GSM1447330.

*Mean replication timing data*

Mean replication timing (MRT) was derived from RepliSeq data [6, 68] for 100 kb non-overlapping windows in hg18 coordinates. The MRT profile of Gm06990 was obtained from the authors [87].

*Gene expression data*

For the Additional file 1: Figure S13, expression data were retrieved from the Genome Browser of the University of California Santa Cruz (UCSC). We downloaded expression values from the release 2 of Caltech RNA-seq track (ENCODE project at UCSC, http://hgdownload.cse.ucsc.edu/goldenPath/hg18/encodeDCC/wgEncodeCalthechRnaSeq/).

Expression for one gene is given in reads per kb of exon model per million mapped reads (RPKM) [88]. RPKM is defined as:

5 $$  R=\frac{10^{9}C}{NL} \; \;,  $$

where *C* is the number of mappable reads that fall into gene exons (union of exons for genes with alternative splicing), *N* is the total number of mappable reads in the experiment, and *L* is the total length of the exons in base pairs. We associated 17 872 genes with a valid RPKM value in Gm12878.

To define the genes and the intergenes regions in Table 1, as well as the totally intergenic 100 kb windows, we determined the genome coordinates of each gene (labeled by its RefSeq identifier) by using RefSeq Genes track. For genes associated with more than one splicing variant, we merged exons coordinates by taking their union. We obtained a table of 23 329 genes. The intergenes coordinates correspond to intervals between genes from which we remove 2 kb from each side (to be sure to be in an ‘intergenic’ region). For the Additional file 1: Figure S12, we used the transcription start site (TSS) associated to these genes (placed at the beginning of the first exon).

*Euchromatin and heterochromatin regions*

Data for the transcriptionally active, early replicating euchromatin regions (*C*1+*C*2 in Gm12878 and *EC*1+*EC*2 in H1hesc) and transcriptionally inactive, late replicating heterochromatin regions (*C*3+*C*4 in Gm12878 and *EC*3+*EC*4 in H1hesc), used in Additional file 1: Figure S15, were obtained from the authors [72, 89].

*Repeat sequence annotations*

We retrieved repeat sequence annotations from the UCSC Genome Browser (rmsk table) obtained using RepeatMasker [90].

## Abbreviations

BGC, biased gene conversion; DSB, double strand break; MRT, mean replication timing; MK, McDonald-Kreitman; NDR, nucleosome-depleted region; NIEB, nucleosome-inhibiting energy barrier; NRL, nucleosome repeat-length; RPKM, reads per kb of exon model per million mapped reads; SNP, single nucleotide polymorphisms; TSS, transcription start site; TTS, transcription termination site

## Additional file

Additional file 1 Supplementary Online Material. Additional data file 1 contains supporting **Figures S1** to **S16** and supporting **Tables S1** and **S2** (PDF). (PDF 1802 kb)
