# Supplementary material for: Evidence of selection for an accessible nucleosomal array in human
Source: BMC Genomics. 2016 Jul 29;17:526. doi: 10.1186/s12864-016-2880-2 (PMC4966569; doi:10.1186/s12864-016-2880-2)
Supplement: Additional file 1 — Supplementary Online Material. Additional data file 1 contains supporting Figures S1 to S16 and supporting Tables S1 and S2 (PDF). (PDF 1802 kb) [file 12864_2016_2880_MOESM1_ESM.pdf]

# Supplementary Online Material

## Evidence of selection for an accessible nucleosomal array in human

Guénola Drillon, Benjamin Audit, Françoise Argoul and Alain Arneodo[1]

*Laboratoire de Physique, CNRS, UMR 5672, Université de Lyon, Ecole Normale Supérieure de Lyon, 46 allée d'Italie, 69364 Lyon, France*

[1] **Corresponding author**
E-mail: alain.arneodo@ens-lyon.fr

Supporting Figures S1 to S16

Supporting Tables S1 to S2

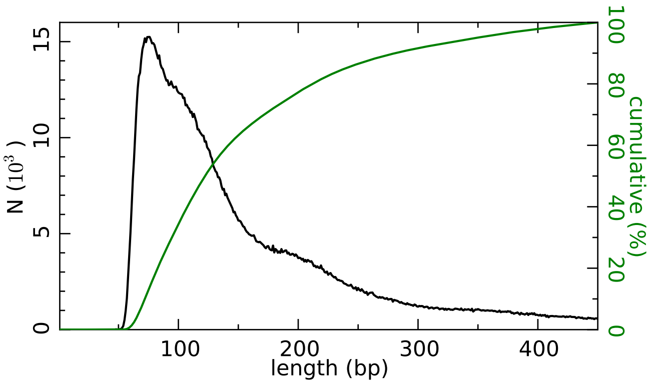


**Figure S1.** Histogram of intrinsic NIEB length (black line) computed from our set of $1,581,256$ NIEBs predicted by the physical model based on sequence-dependent DNA bending properties (mean = 152.5 bp, median = 123 bp) (Materials and Methods). The green curve corresponds to the cumulative histogram.

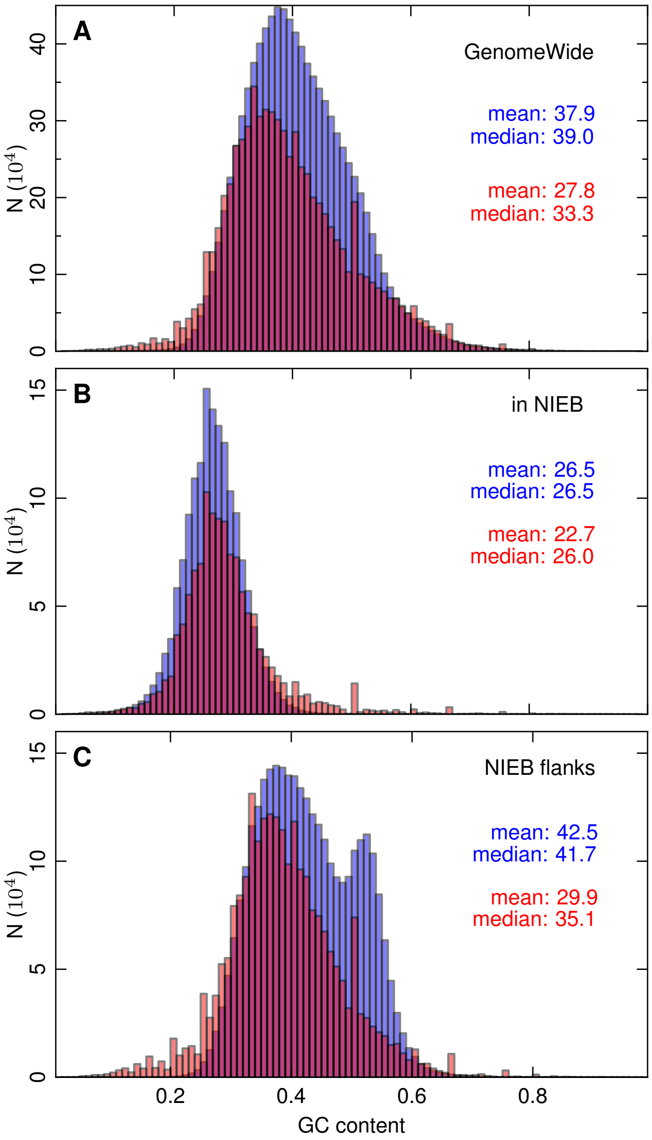


**Figure S2.** Histograms of GC content (blue) computed genome wide in 300 bp non-overlapping windows **(A)**, over the 1,581,256 intrinsic NIEBs **(B)** and over the 2 (on the right and on the left) 300 bp windows flanking these excluding regions **(C)**. The red histograms were computed on the same, but repeat-masked, sequences.

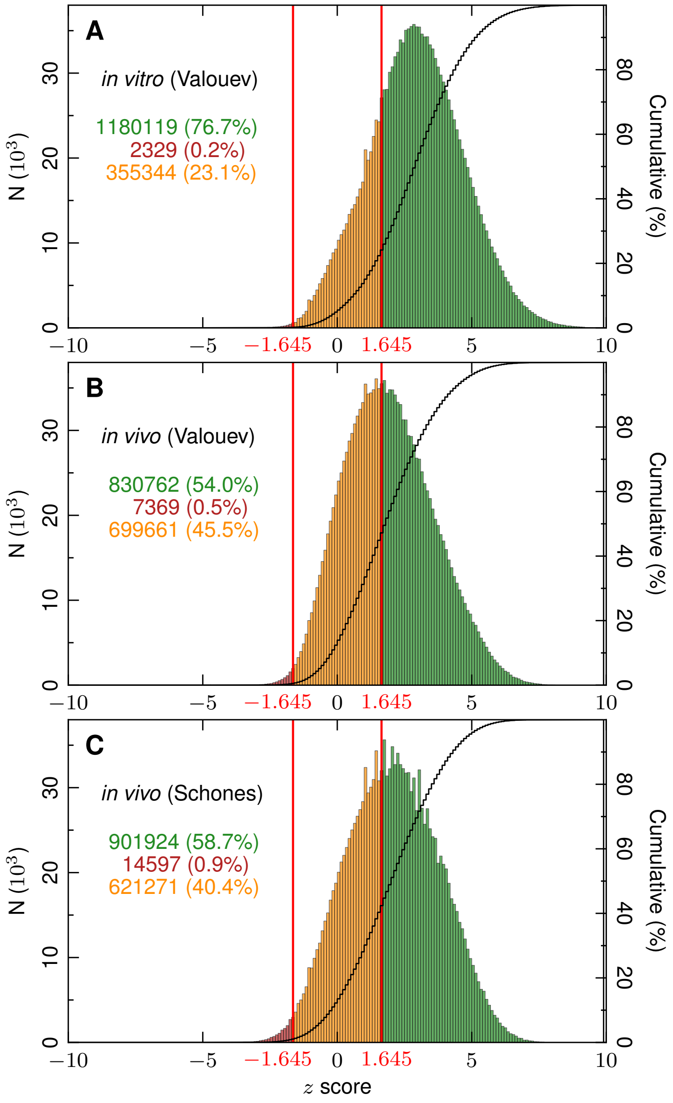


**Figure S3.** Histograms of z-score values in the difference in nucleosome tag densities in flanking 300 bp windows relative to intrinsic NIEBs in G1-G3 groups (see text and Equation (1) in Materials and Methods) for **(A)** "Valouev" *in vitro* data, **(B)** "Valouev" *in vivo* data and **(C)** "Schones" *in vivo* data. Vertical red lines mark the z-value threshold corresponding to the 5 % confidence level for the one-side tests of (i) significant depletion of nucleosome MNase tags in NIEBs (positive threshold) and (ii) significant enrichment of tags NIEBs (negative threshold). Histogram colors correspond to barriers in G1 (green), G2 (red) and G3 (orange). The black curves are the corresponding cumulative histograms.

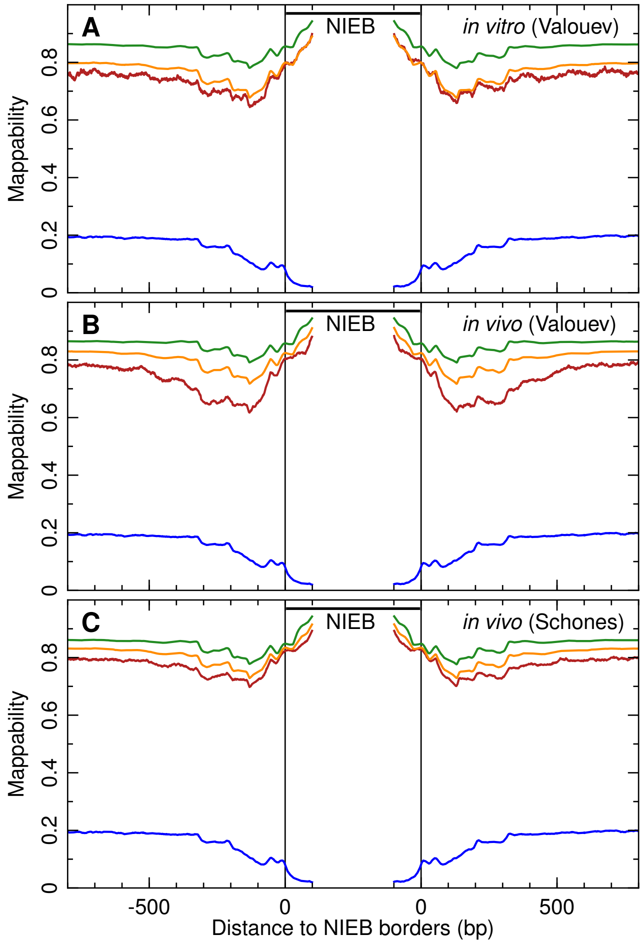


**Figure S4.** Mappability in and around the 1,581,256 predicted NIEBs. The colors correspond to barriers belonging to the class G1 (green), G2 (red), G3 (orange) and to the 43,364 barriers lacking sufficient mappability to perform the test (Supplementary Fig. S3) (blue). **(A)** "Valouev" *in vitro* data: (G1) 1,180,119, (G2) 2,329, (G3) 355,344 barriers. **(B)** "Valouev" *in vivo* data: (G1) 830,762, (G2) 7,369, (G3) 699,661 barriers. **(C)** "Schones" *in vivo* data: (G1) 901,924, (G2) 14,597, (G3) 621,271 barriers.

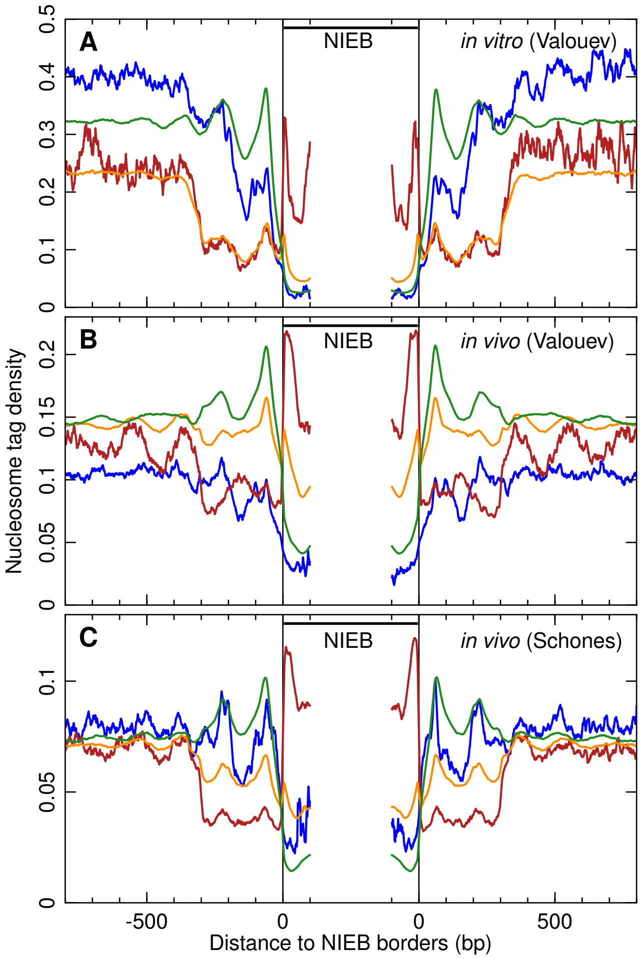


**Figure S5.** Nucleosome tag density on both sides of the 1, 581, 256 predicted NIEBs after taking into account the experimental mappability (Supplementary Fig. S4) (Materials and Methods). **(A)** "Valouev" *in vitro* data; **(B)** "Valouev" *in vivo* data; **(C)** "Schones" *in vivo* data. Curves were smoothed over 10 bp windows. The colors have the same meaning as in Supplementary Figure S4.

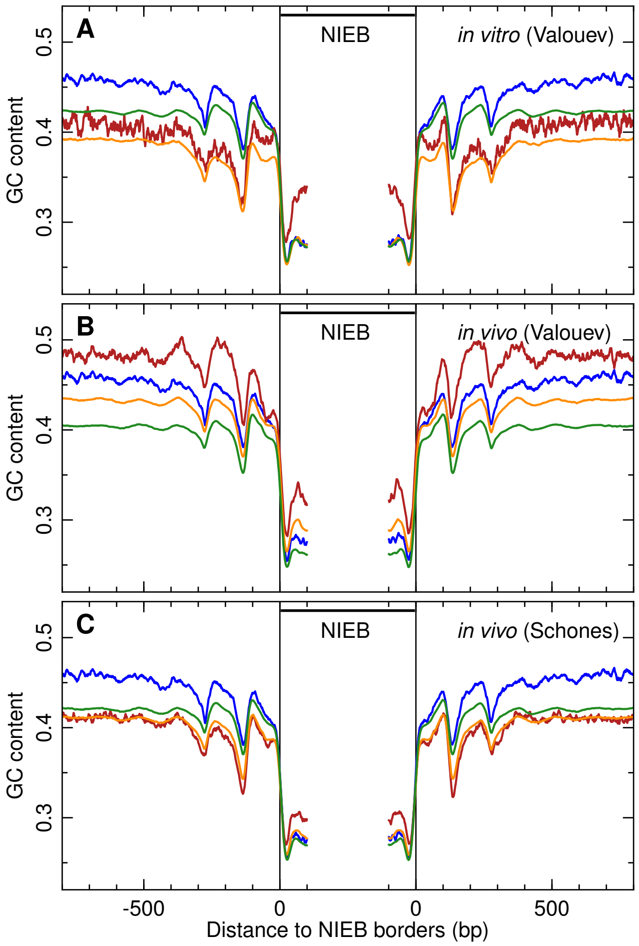


**Figure S6.** Mean GC content on both sides of the 1,581,256 predicted NIEBs. The colors have the same meaning as in Supplementary Figure S4. **(A)** "Valouev" *in vitro* data; **(B)** "Valouev" *in vivo* data; **(C)** "Schones" *in vivo* data. Curves were smoothed over 10 bp windows.

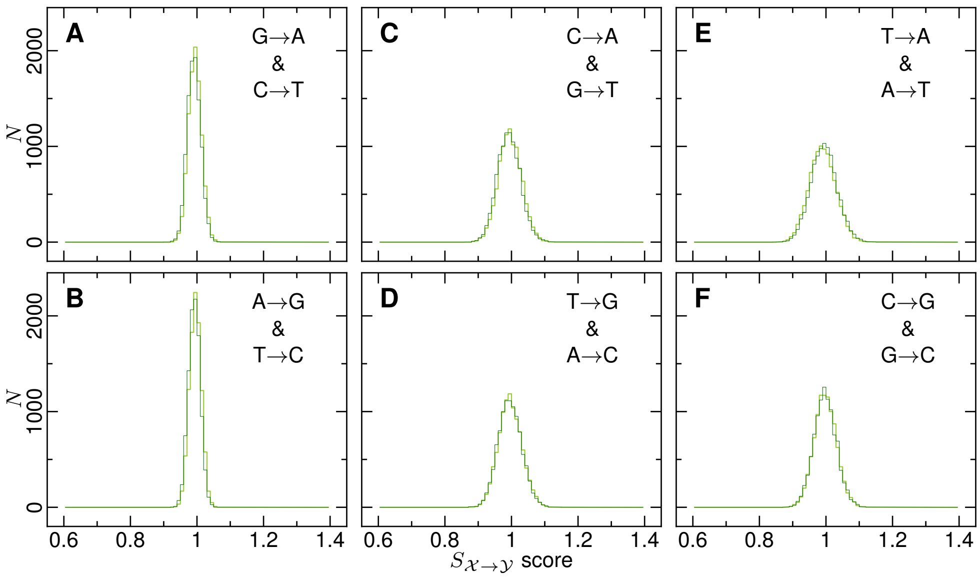


**Figure S7.** Distribution of $S_{\mathcal{X}\to\mathcal{Y}}$ ratios under the hypothesis of neutral evolution. Neutral $S_{\mathcal{X}\to\mathcal{Y}}$ values were obtained by sampling one 10 bp in the center of each large inter-NIEB ($l > 800$ bp). Histograms (bin size 0.01) of 10000 realizations of this process were computed (Methods). The pannels correspond to the substitution rates: **(A)** G $\to$ A and C $\to$ T; **(B)** A $\to$ G and T $\to$ C; **(C)** C $\to$ A and G $\to$ T; **(D)** T $\to$ G and A $\to$ C; **(E)** T $\to$ A and A $\to$ T; **(F)** C $\to$ G and G $\to$ C. In each panel the first (resp. second) substitution is represented in dark green (resp. light green).

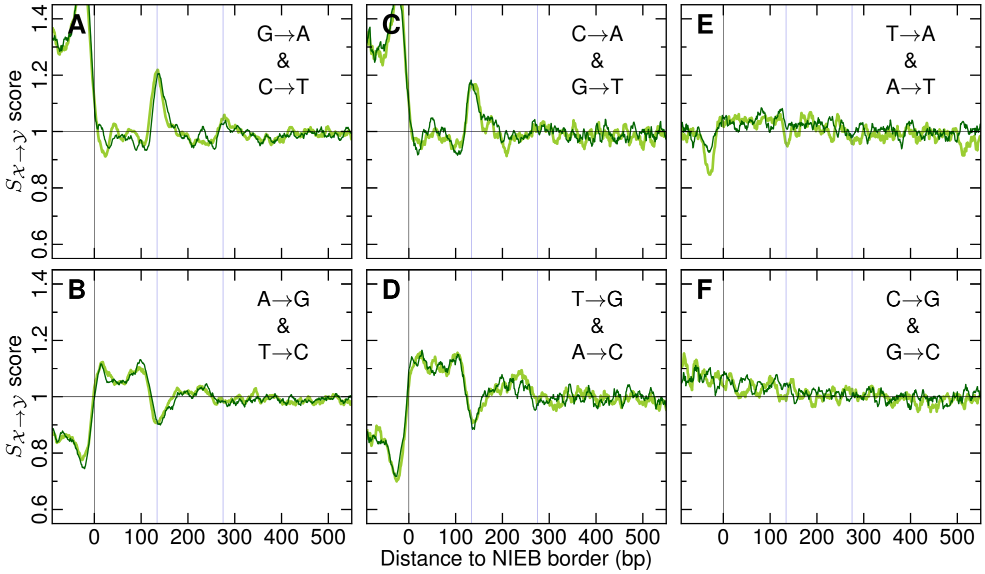


**Figure S8.** Evidence of selection in and around the 1, 581, 256 NIEBs for all intraspecies single nucleotide polymorphisms including those with a minor allele frequency less than 1%. Ratios $S_{\mathcal{X}\to\mathcal{Y}}$ of background corrected inter- and intraspecies divergence rates plotted against the position from the closest NIEB border (negative distances correspond to loci inside the NIEBs). The pannels correspond to the subsitution rates: **(A)** G $\to$ A and C $\to$ T, **(B)** A $\to$ G and T $\to$ C, **(C)** C $\to$ A and G $\to$ T, **(D)** T $\to$ G and A $\to$ C, **(E)** T $\to$ A and A $\to$ T, **(F)** C $\to$ G and G $\to$ C. In each panel the first (resp. second) substitution is represented in dark green (resp. light green). Curves were smoothed over 10 bp windows. The vertical blue lines have the same meaning as in Figure 1A'-C'.

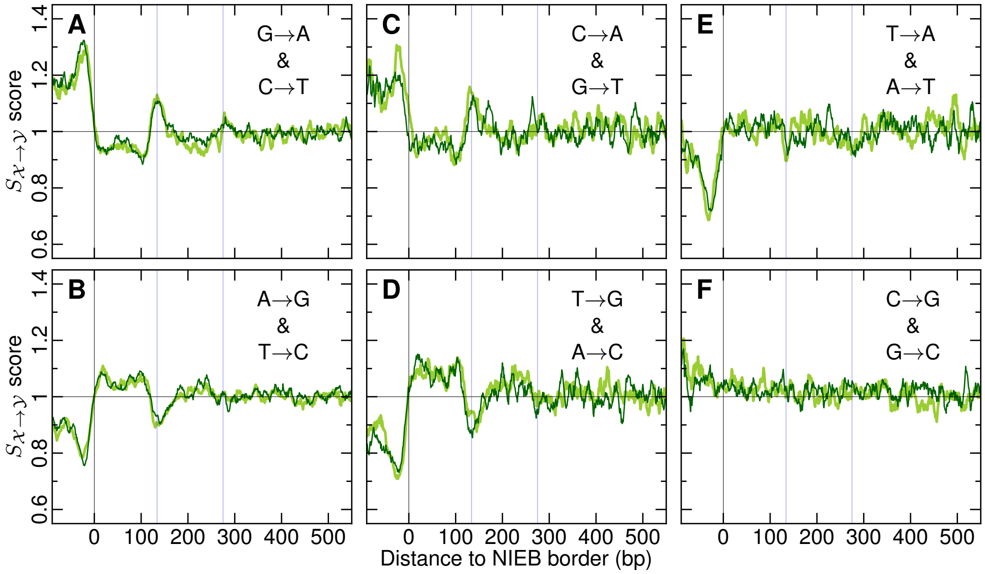


**Figure S9.** Evidence of selection in and around the 1,581,256 NIEBs after removing repeated sequences prior to our analysis (Materials and Methods). Ratios $S_{\mathcal{X}\to\mathcal{Y}}$ of background corrected inter- and intraspecies divergence rates plotted against the position from the closest NIEB border (negative distances correspond to loci inside the NIEBs). The pannels correspond to the subsitution rates: **(A)** G → A and C → T, **(B)** A → G and T → C, **(C)** C → A and G → T, **(D)** T → G and A → C, **(E)** T → A and A → T, **(F)** C → G and G → C. In each panel the first (resp. second) substitution is represented in dark green (resp. light green). Curves were smoothed over 10 bp windows. The vertical blue lines have the same meaning as in Figure 1A'-C'.

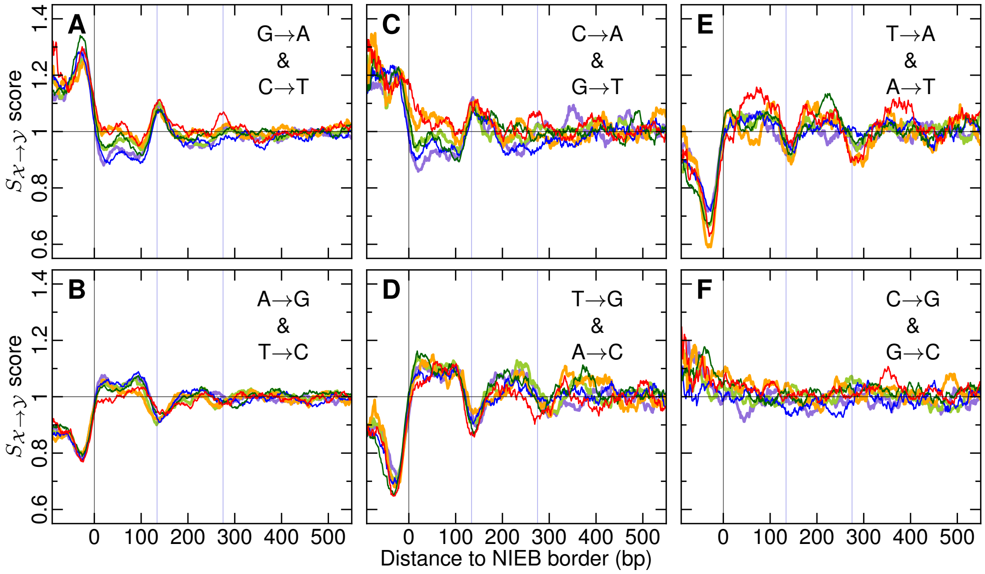


**Figure S10.** Ratios $S_{\mathcal{X}\to\mathcal{Y}}$ of background corrected inter- and intraspecies divergence rates plotted against the position from the closest NIEB border (negative distances correspond to loci inside the NIEBs), for the three classes of NIEBs defined by their local reference GC content (Fig. 7C,D): GC $< 0.38$ (blue, purple), $0.38 \leq$ GC $< 0.46$ (dark green, light green) and $0.46 \leq$ GC (red, orange). **(A)** G $\to$ A and C $\to$ T; **(B)** A $\to$ G and T $\to$ C; **(C)** C $\to$ A and G $\to$ T; **(D)** T $\to$ G and A $\to$ C; **(E)** T $\to$ A and A $\to$ T; **(F)** C $\to$ G and G $\to$ C. In each panel the first (resp. second) substitution is represented in blue, dark green, red (resp. purple, light green, orange). Curves were smoothed over 30 bp windows. The vertical blue lines have the same meaning as in Figure 1A'-C'.

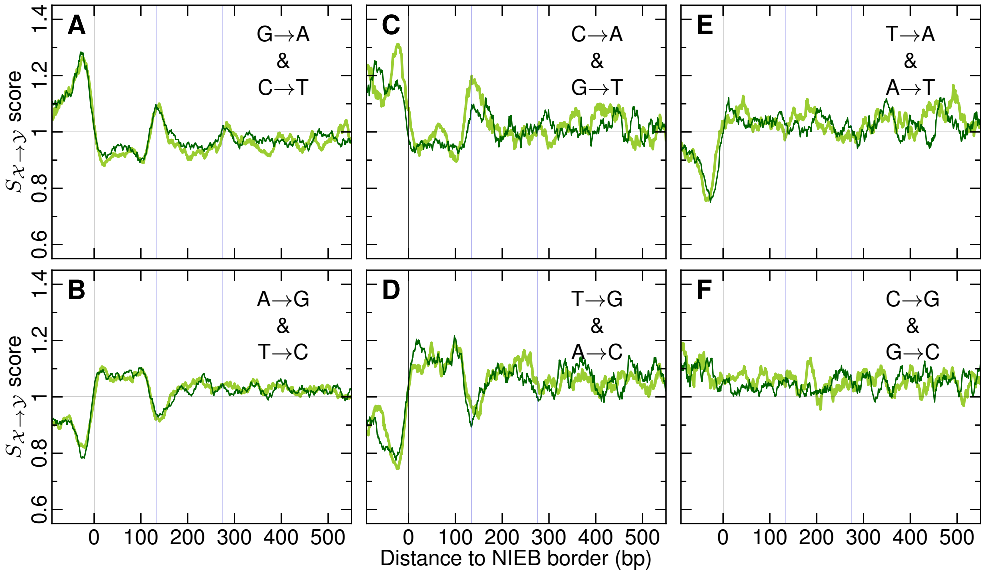


**Figure S11.** Evidence of selection in and around the 790, 285 NIEBs found in totally intergenic 100 kb windows. Ratios $S_{\mathcal{X}\to\mathcal{Y}}$ of background corrected inter- and intraspecies divergence rates plotted against the position from the closest NIEB border (negative distances correspond to loci inside the NIEBs). The pannels correspond to the subsitution rates: **(A)** G → A and C → T, **(B)** A → G and T → C, **(C)** C → A and G → T, **(D)** T → G and A → C, **(E)** T → A and A → T, **(F)** C → G and G → C. In each panel the first (resp. second) substitution is represented in dark green (resp. light green). Curves were smoothed over 20 bp windows. The vertical blue lines have the same meaning as in Figure 1A'-C'.

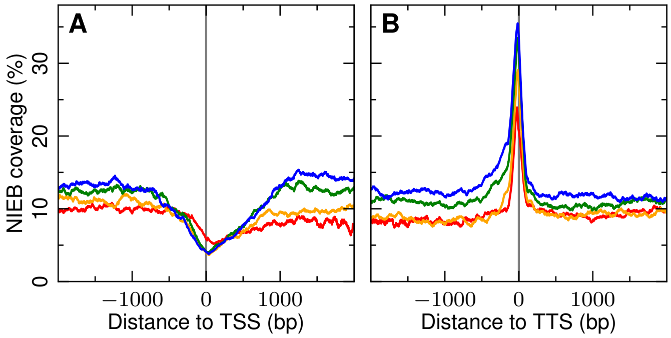


**Figure S12.** Mean profiles of NIEB coverage around human gene TSS **(A)** and TTS **(B)**. The different coloured profiles correspond to average over genes of length $L < 5$ kb (red; 5, 969), $5 \leq L < 19$ kb (orange; 6, 082), $19 \leq L < 54$ kb (green; 5, 752) and $L \geq 54$ kb (blue; 5, 969).

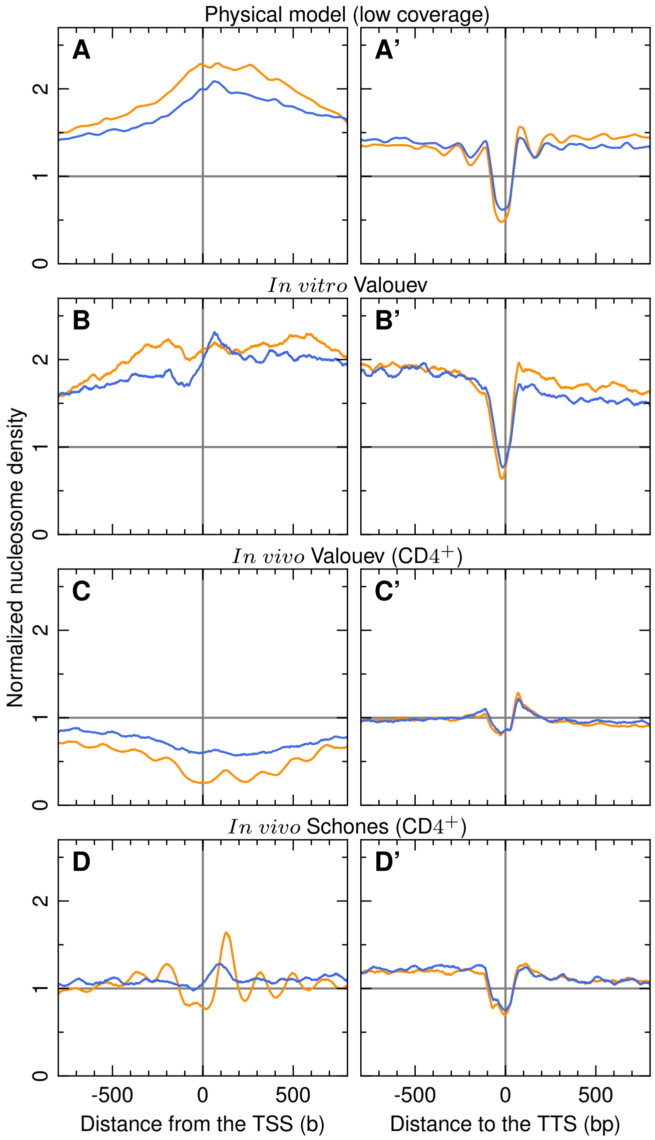


**Figure S13.** Normalized (with respect to the genome average) nucleosome density profiles around human gene TSS (resp. TTS) computed with the sequence-dependent physical model at low genomic nucleosome average **(A)** (resp. **(A')**) and obtained with "Valouev" *in vitro* **(B)** (resp. **(B')**), "Valouev" *in vivo* **(C)** (resp. **(C')**) and "Schones" *in vivo* **(D)** (resp. **(D')**) experimental data (Materials and Methods). The different coloured profiles correspond to average over $9,372$ genes expressed in Gm12878 (orange) and $8,500$ genes non-expressed in Gm12878 (blue) (Materials and Methods).

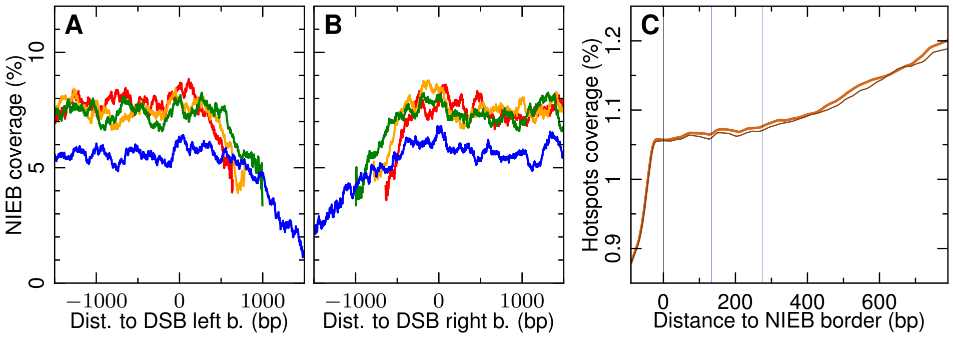


**Figure S14.** Mean profiles of NIEB coverage around double strand break (DSB) hotspots in human individuals (Materials and Methods). **(A)** Mean profiles of the left (5'-) side of the DSB hotspots. **(B)** Mean profiles of the right (3'-) side of the DSB hotspots. In (A) (resp.(B)), the origin stands for the left (resp. right) DSB hotspots border. The different coloured profiles correspond to average over DSB hotspots of length $l < 1.3$ kb (red), $1.3 \leq l < 1.7$ kb (orange), $1.7 \leq l < 2.0$ kb (green) and $l \geq 2.0$ kb (blue). **(C)** Mean DSB hotspot coverage profiles inside (distance to NIEB border $< 0$) and nearby (distance to NIEB border $> 0$) the $1,581,256$ intrinsic NIEBs. The colors correspond to the mean profiles obtained to the 5' NIEB border (brown) and from the 3' NIEB border (light brown).

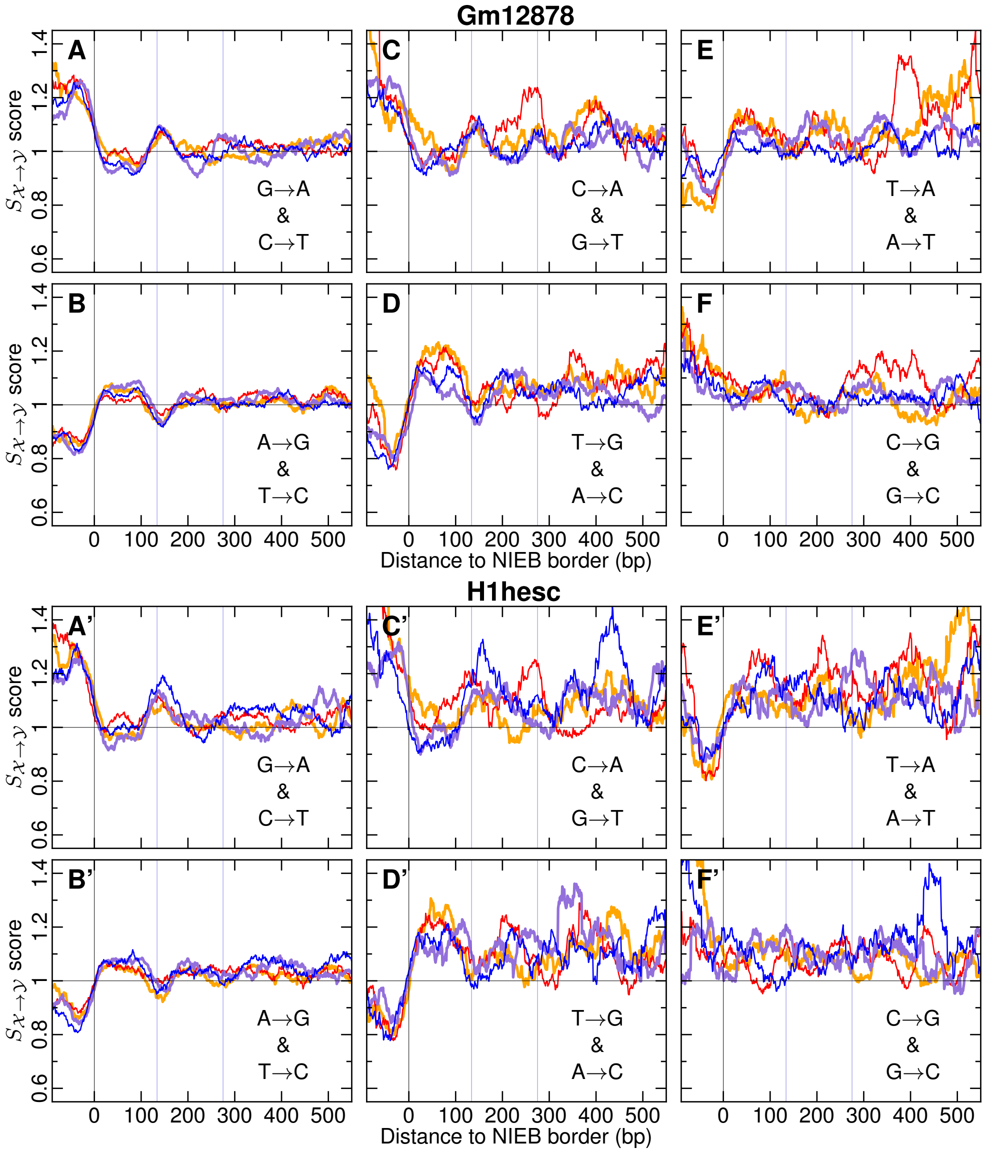


**Figure S15.** Evidence of selection in and around the NIEBs belonging to transcriptionally active, early replicating $(C1 + C2)$ (resp. $(EC1 + EC2)$) euchromatin regions (red, orange; $n = 219,191$) (resp. $n = 173,621$) and to transcriptionally inactive, late replicating $(C3 + C4)$ heterochromatin (resp. $(EC3 + EC4)$) regions (blue, purple; $n = 208,180$) (resp. $n = 95,447$) in Gm12878 (resp. H1hesc) human cell type (Materials and Methods). Ratios $S_{X\to Y}$ of background corrected inter- and intraspecies divergence rates plotted against the position from the closest NIEB border (negative distances correspond to loci inside the NIEBs). The pannels correspond to the subsitution rates: **(A)** (resp. **(A')**) G $\to$ A and C $\to$ T, **(B)** (resp. **(B')**) A $\to$ G and T $\to$ C, **(C)** (resp. **(C')**) C $\to$ A and G $\to$ T, **(D)** (resp. **(D')**) T $\to$ G and A $\to$ C, **(E)** (resp. **(E')**) T $\to$ A and A $\to$ T, **(F)** (resp. **(F')**) C $\to$ G and G $\to$ C. In each panel the first (resp. second) substitution is represented in (red, blue) (resp. (orange, purple)). Curves were smoothed over 50 bp windows. The vertical blue lines have the same meaning as in Figure 1A'-C'.

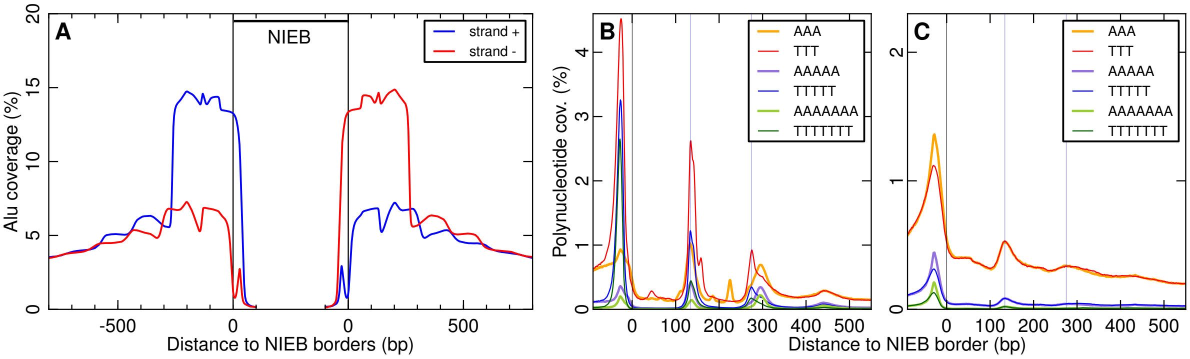


**Figure S16.** **(A)** Mean profile of sense (blue) and antisense (red) Alu elements on both sides of the 1, 581, 256 predicted NIEBs. **(B)** (resp. **(C)**) Mean profile of polynucleotide coverage in and around the 729, 641 (resp. 2, 432, 871) NIEB borders that are (resp. are not) flanked by an Alu element: AAA (orange), TTT (red), AAAAA (purple), TTTTT (blue), AAAAAAA (light green), TTTTTTT (dark green) (Materials and Methods).

**Table S1.** Large-scale distribution of the predicted $1,581,256$ NIEBs along the 22 human autosomes.

| | (% 100 kb windows) | NIEB/kb | cov (%) |
|---|---|---|---|
| allNDR | 47.4 | 0.56 | 9.0 |
| L1 (G+C $<$ 38 %) | 24.6 | 0.54 | 9.4 |
| L2 (38 $\leq$ G+C $<$ 42 %) | 14.5 | 0.59 | 9.1 |
| H1 (42 $\leq$ G+C $<$ 47 %) | 6.4 | 0.59 | 8.1 |
| H2 (47 $\leq$ G+C $<$ 52 %) | 1.5 | 0.49 | 6.0 |
| H3 (52 % $\leq$ G+C) | 0.4 | 0.37 | 4.3 |
| Early (MRT $<$ 0.36) | 5.4 | 0.63 | 9.2 |
| Medium (0.36 $\leq$ MRT $<$ 0.69) | 18.9 | 0.57 | 8.8 |
| Late (0.69 $\leq$MRT) | 23.2 | 0.54 | 9.1 |
| Low DNase ($<$ 14.3 reads/kb) | 24.3 | 0.53 | 9.1 |
| Medium DNase | 19.1 | 0.59 | 9.1 |
| High DNase (29.3 reads/kb $<$) | 3.9 | 0.58 | 8.2 |
| Low Recomb ($<$ 0.378 cM/Mb) | 14.4 | 0.55 | 9.3 |
| Medium Recomb | 19.6 | 0.56 | 9.0 |
| High Recomb (1.681 cM/Mb $<$) | 13.5 | 0.57 | 8.6 |

NOTE. - NIEB density was computed in $12,373$ totally intergenic 100 kb non-overlapping windows that were classified according to their GC content, MRT, DNase I sensitivity raw tag density and meiotic recombination rate (Materials and Methods). The first column defines the region, the second column its corresponding genome coverage (%), the third column the mean NIEB density (NIEB/kb) and the fourth column, the corresponding coverage of this region by the set of NIEBs (%). For each row, we estimated the standard error of the mean NIEB density to be SEM $<$ 0.015.

**Table S2.** Large-scale distribution of the $1,581,005$ inter-NIEB regions along the 22 human autosomes.

| | | 1 nuc | 2 nuc | 3 nuc | 4 nuc | 5 nuc | > 5 nuc |
|---|---|---|---|---|---|---|---|
| | (%) | 9.2 | 9.4 | 7.4 | 7.4 | 6.9 | 59.7 |
| L1 (G+C < 38 %) | 33.3 | 0.88 | 0.73 | 0.80 | 0.80 | 0.84 | 0.96 |
| L2 (38 ≤ G+C < 42 %) | 32.3 | 1.08 | 1.04 | 1.06 | 1.06 | 1.07 | 1.02 |
| H1 (42 ≤ G+C < 47 %) | 21.4 | 1.10 | 1.25 | 1.19 | 1.18 | 1.14 | 1.01 |
| H2 (47 ≤ G+C < 52 %) | 9.2 | 0.83 | 1.06 | 0.93 | 0.95 | 0.90 | 0.91 |
| H3 (52 % ≤ G+C) | 3.7 | 0.63 | 0.82 | 0.67 | 0.71 | 0.63 | 0.77 |
| Early (MRT < 0.36) | 30.0 | 1.11 | 1.30 | 1.21 | 1.21 | 1.14 | 0.99 |
| Medium (0.36 ≤ MRT < 0.69) | 40.0 | 0.96 | 0.92 | 0.95 | 0.95 | 0.96 | 0.98 |
| Late (0.69 ≤MRT) | 30.0 | 0.86 | 0.72 | 0.79 | 0.78 | 0.84 | 0.95 |
| Low DNase (< 14.3 reads/kb) | 30.0 | 0.85 | 0.69 | 0.76 | 0.76 | 0.81 | 0.95 |
| Medium DNase | 40.0 | 1.00 | 0.97 | 1.00 | 1.00 | 1.00 | 1.00 |
| High DNase (29.3 reads/kb <) | 30.0 | 1.07 | 1.26 | 1.17 | 1.17 | 1.10 | 0.97 |
| Low Recomb (< 0.378 cM/Mb) | 30.0 | 1.05 | 1.06 | 1.04 | 1.05 | 1.02 | 0.97 |
| Medium Recomb | 40.0 | 0.96 | 0.94 | 0.95 | 0.95 | 0.96 | 0.98 |
| High Recomb (1.681 cM/Mb <) | 30.0 | 0.92 | 0.94 | 0.95 | 0.93 | 0.95 | 0.98 |
| Genes | 47.1 | 1.03 | 1.07 | 1.06 | 1.06 | 1.04 | 0.99 |
| Intergenes | 51.6 | 0.95 | 0.91 | 0.93 | 0.93 | 0.95 | 0.99 |

NOTE. - These inter-NIEB regions were defined according to their length $d$ as crystal-like regions ($d \leq 800$ bp) containing $n$ (from 1 to 5) well positioned nucleosomes and larger ($d > 800$ bp) regions with fuzzy nucleosome positioning at the center. They were further classified according to their GC content, MRT, DNase I sensitivity raw tag density, meiotic recombination rate and gene and intergene location (Materials and Methods). The first row indicates the proportion of each of these 5 crystal-like regions and the larger ones over the $1,581,005$ inter-NIEB regions. The first column indicates the genome coverage (%) of the different classes. Other numbers corresponds to ratio between the observed number of crystal-like regions belonging to a specific classes over the expected one (proportional to the coverage of the different classes).
